# Effects of Polyphenols on Oxidative Stress, Inflammation, and Interconnected Pathways during Spinal Cord Injury

**DOI:** 10.1155/2022/8100195

**Published:** 2022-01-07

**Authors:** Sajad Fakhri, Fatemeh Abbaszadeh, Seyed Zachariah Moradi, Hui Cao, Haroon Khan, Jianbo Xiao

**Affiliations:** ^1^Pharmaceutical Sciences Research Center, Health Institute, Kermanshah University of Medical Sciences, Kermanshah 6734667149, Iran; ^2^Department of Neuroscience, Faculty of Advanced Technologies in Medical Sciences, Iran University of Medical Sciences, Tehran, Iran; ^3^Neurobiology Research Center, Shahid Beheshti University of Medical Sciences, Tehran, Iran; ^4^Medical Biology Research Center, Health Technology Institute, Kermanshah University of Medical Sciences, Kermanshah 6734667149, Iran; ^5^Nutrition and Bromatology Group, Department of Analytical Chemistry and Food Science, Faculty of Food Science and Technology, University of Vigo-Ourense Campus, E-32004 Ourense, Spain; ^6^Department of Pharmacy, Abdul Wali Khan University Mardan, 23200, Pakistan; ^7^Institute of Food Safety and Nutrition, Jinan University, Guangzhou 510632, China

## Abstract

Despite the progression in targeting the complex pathophysiological mechanisms of neurodegenerative diseases (NDDs) and spinal cord injury (SCI), there is a lack of effective treatments. Moreover, conventional therapies suffer from associated side effects and low efficacy, raising the need for finding potential alternative therapies. In this regard, a comprehensive review was done regarding revealing the main neurological dysregulated pathways and providing alternative therapeutic agents following SCI. From the mechanistic point, oxidative stress and inflammatory pathways are major upstream orchestras of cross-linked dysregulated pathways (e.g., apoptosis, autophagy, and extrinsic mechanisms) following SCI. It urges the need for developing multitarget therapies against SCI complications. Polyphenols, as plant-derived secondary metabolites, have the potential of being introduced as alternative therapeutic agents to pave the way for treating SCI. Such secondary metabolites presented modulatory effects on neuronal oxidative stress, neuroinflammatory, and extrinsic axonal dysregulated pathways in the onset and progression of SCI. In the present review, the potential role of phenolic compounds as critical phytochemicals has also been revealed in regulating upstream dysregulated oxidative stress/inflammatory signaling mediators and extrinsic mechanisms of axonal regeneration after SCI in preclinical and clinical studies. Additionally, the coadministration of polyphenols and stem cells has shown a promising strategy for improving post-SCI complications.

## 1. Introduction

As pivotal causes of disability and death, neurological diseases (NDDs) increasingly affect millions of people worldwide. Despite the advances in revealing multiple dysregulated pathways in the pathogenesis of NDDs, their main pathophysiological mechanisms have remained to be elucidated [1, 2]. Spinal cord injury (SCI) is a NDD with sensory-motor dysfunctionalities, which extremely reduces the quality of life. The worldwide prevalence of SCI is on the rise, more in 14.6-67.6-year-old individuals and four times more prevalent in men [3, 4]. In the United States, the annual incidence of traumatic SCI is estimated at about 12,000 new cases per year, equates to 40 cases per million of population, and bears high costs [5].

From the pathophysiological point of view, SCI involves primary and secondary phases. While the primary injury occurs after the spinal mechanical trauma, the secondary phase consists of intrinsic oxidative stress, inflammatory, apoptotic, and autophagic cascades [6]. On the other hand, extrinsic pathways also play critical roles in the progression of SCI, including glial scar formation and axonal degeneration [7]. Of intrinsic pathways, oxidative stress, neuroinflammation, and neuroapoptosis are interconnected with extrinsic pathways (e.g., axonal signaling). Thus, the aforementioned pathological pathways play destructive roles in neuronal cell death and neurodegenerative processes, leading to cell death. The modulatory effects of antioxidant defenses on neuroinflammatory and neuroapoptotic responses play key roles in the onset and progression of SCI via affecting microglia, astrocytes, and associated mediators [8, 9].

Despite progressions in clinical healthcare, prevention/treatment of SCI has remained a clinical challenge. This challenge could be due to the complex pathophysiological mechanisms of SCI, which raises the need for developing novel multitarget therapeutics. Such treatments could target both the intrinsic and extrinsic pathways [3].

It is worth mentioning that providing new drugs from plants has a long and successful history in complementary and alternative medicine. In this line, the plant kingdom has shown promising results against SCI. Among natural entities, polyphenols/phenolic compounds are multitarget and accessible phytochemicals with high specificity and low toxicity levels. They are currently used in modern medicine to design and develop new therapeutic agents because of their extensive biological activities and health benefits. These compounds have been considered safe dietary agents with potential inhibitory effects against oxidative stress and inflammation in various NDDs [3, 10]. In recent years, growing evidence has focused on the administration of natural neuroprotective polyphenols with potential antioxidant activities to manage SCI and other NDDs [11].

Previous reviews indicated the crucial role of oxidative stress and axonal dysregulations in the pathogenesis of Parkinson's disease [11], epilepsy [12], and some other NDDs [13]. Accordingly, cytokines [14], nitric oxide (NO), antioxidant response element (ARE) [15], and other oxidative stress/inflammatory mediators, as well as extrinsic dysregulated pathways, seem to be potential regulators of such pathological conditions. We have also previously provided the role of apoptosis and autophagy following SCI [3]; however, no focus has been yet done on the pivotal role of oxidative stress after SCI. Therefore, the present study comprehensively reviews oxidative stress pathways and their effects on the inflammatory and apoptotic pathways during SCI. The modulatory effects of polyphenols/phenolic compounds on the intrinsic upstream oxidative stress pathways and extrinsic axonal pathways have also been revealed following SCI. Besides, the coadministration of polyphenols and stem cells has been shown as a new gate in combating post-SCI complications.

## 2. The Role of Oxidative Stress, Inflammation, and Apoptosis after SCI: Intrinsic Pathway

Oxidative stress has been described as a disturbance and perturbation in the balance among the production and manifestation of reactive oxygen species (ROS) and the biological antioxidant defense ability. It causes toxic effects, tissue injury, inflammation, apoptosis, autophagy, and necrosis via the generation of free radicals and peroxides, which damages DNA, proteins, lipids, and carbohydrates [16]. ROS presuppose both the free radical and nonfree radical oxygen moderators like superoxide, hydrogen peroxide (H_2_O_2_), hydroxyl radical (^·^OH), and singlet oxygen. Even though ROS could operate as redox signaling messengers, oxidative stress can also cause perturbation in normal cellular signaling mechanisms. From the latter point, oxidative stress is considered one of the most important factors in variant abnormality conditions such as cancer, chronic obstructive pulmonary disease, cardiovascular disease, chronic kidney disease, and NDDs [17, 18].

Nowadays, SCI has been a critical health problem among NDDs. The pathophysiology of SCI is defined through a primary injury/initial trauma, followed by the second cascades of injuries that are consequences of the indirect achievement of the primary injury. The secondary injury continues at different time points after the primary injury and is not restricted to the same primary injury site. Oxidative stress is one of the critical components of the later phase, which is receiving increasing attention. Many studies have been performed to investigate the destructive activity of oxidative stress after SCI and its confounding effects on improving the functional recovery following SCI [19].

Various factors predispose the neurons of the central nervous system to electrophilic/oxidative stress. Among those factors, polyunsaturated fatty acids (e.g., arachidonic acid and linoleic acid), low antioxidant capacity, reactive oxygen metabolites, and neural mitochondrial changes are the critical ones [9, 20]. Decreasing glutathione (GSH) and the levels of copper/zinc superoxide dismutase (SOD), as well as increasing malondialdehyde (MDA), and acrolein are considered critical hallmarks of oxidative stress after SCI [9]. In parallel, the mechanical injury detected in the plasma membrane resulting from SCI leads to the disruption of normal ion balance and obviously to the enhancement of the intracellular concentration of sodium and calcium. Besides oxidative stress and increasing glutamate (Glu), there are other aggravating factors for increasing intracellular calcium. Consequently, Na^+^/K^+^ ATPase works to improve the electrochemical gradient of the neuronal cell membrane via decreasing the intracellular sodium that leads to reversing the activity of the Na^+^/Ca^2+^ exchanger, thereby removing sodium and increasing the intracellular concentration of calcium and related oxidative toxicity. Increased intracellular calcium causes mitochondrial damage and ROS production and activates phospholipase, caspases, and proteases, followed by an increase in the release of membrane fatty acids [20]. Also, SCI impairs ATP production, recruits neutrophils to the injury site, stimulates xanthine oxidase (XO), and produces more ROS [21]. In the same way, lipid peroxidation (LPO) can increase oxidative stress via the generation of more free radicals and reactive aldehydes [20]. Since oxidative stress and ROS are key elements in the pathophysiology of SCI, reduction of oxidative stress is considered one of the possible effective mechanisms in the treatment/improvement of SCI patients. In recent years, several studies have investigated the antioxidative effects of various bioactive compounds in animal models of SCI.

In addition to oxidative stress, inflammatory responses are essential components of SCI secondary injury that play significant roles in regulating the pathogenesis of acute and chronic phases of SCI and play a special role in nerve injury and regenerative responses [22]. After SCI, ruptured blood vessels disturb the blood-brain barrier and infiltrate the neutrophils to the injury site. The proliferation or migration of activated glial and peripherally derived immune cells initiates the inflammatory responses in the central nervous system after SCI. T cells are one of the main pillars of the inflammatory and immune response and necessary factors for mounting macrophages in these processes [22]. Also, the activated myelin basic protein- (MBP-) reactive T cells lead to transient paralysis and neuroinflammation in SCI. Microglia and macrophages are involved in the inflammatory response and secondary pathology via facilitating the release of cytokines, interferon, interleukin- (IL-) 1, IL-6, IL-10, and tumor necrosis factor (TNF), and the activation of interleukin receptors (IL-2R and IL-4R). Furthermore, cytokines produce inflammatory responses in the central nervous system by enhancing the expression of additional reactive oxygen, cytokines, NO, and chemokines [22]. After SCI, IL-6 and TNF-*α* are upregulated around the damaged area. It was documented that TNF-*α* could increase the neuronal cell death mediated by Glu in the rat spinal cord and TNF-*α* antagonists decrease the development and extension of inflammation as well as tissue injury events dependent on SCI. Suspiciously similar treatment with the IL-6 receptor monoclonal antibody diminishes the differentiation of astrocytes, reduces the number of inflammatory cells, and detracts the formation of connective tissue scars [22]. Cytokines showed an important role in SCI-induced inflammatory processes, and enhancing the production of IL-1 family cytokines (like IL-1*α*) is considered a vital trigger for this process. IL-1*α* and IL-18 are known as intense mediators of inflammation that can initiate and/or augment an extensive type of effects associated with host responses to tissue injury and innate immunity. SCI induces the activation of caspase-1, enhances the operation of IL-1*α* and IL-18, and cleaves the X-linked inhibitors of apoptosis protein (XIAP) [22]. Accordingly, decreasing inflammation could modulate the functional deficit and secondary degeneration after SCI.

Apoptosis is another important physiological process for removing cells, and its implementation is necessary for the regular function and development of multicellular organisms. Apoptosis signaling occurs via several pathways inchoate from triggering events within or outside the cell [23]. Two main pathways are known for the mechanism of cellular apoptosis. During the first pathway, mitochondrial dysfunction begins with different triggers such as ATP depletion or hypoxia, leading to the release of cytochrome from mitochondria and caspase-9 activation. On the other hand, the extrinsic apoptosis pathway is associated with a group of cell surface receptors known as cell death receptors (DRs) and belongs to the TNF receptor (TNFR) family. TNFR consists of Fas, Fas ligand, TNFR1, DR3, and p75, leading to the activation of caspase-8 and caspase-10 via their intracellular death domains [8, 9]. Caspase-9 and caspase-8 are the major caspases in mitochondrial-dependent pathways and the DR, respectively, which regulate the process; downstream effector caspases (as caspase-3) are activated through proteolytic cleavage and initiator caspases. The Bcl-2 family of proteins, p21-activated kinase, caspase-activated DNase inhibitor, cytoskeletal proteins (such as gelsolin), proteins involved in mRNA splicing, DNA repair, DNA replication, and focal adhesion kinase are some essential proteins among the identified target substrates for caspase-3 [22, 24]. Following SCI, the Fas and p75 receptors are expressed on the spinal cord's microglia, astrocytes, and oligodendrocytes. Some of the extracellular excitatory amino acids such as Glu can increase glial and neuronal cell death's delayed apoptosis via the activation of the N-methyl-D-aspartate (NMDA) receptor. Although SCI's exact mechanism remains unclear, calcium-dependent and TNF-*α*-mediated activation, inducible nitric oxide synthase (iNOS), and NO production are possible factors involved in this process, and it is proven that Glu-induced cell death was amplified by TNF-*α*. Also, TNF-*α* activates the nuclear factor kappa-light-chain-enhancer of activated B cells (NF-*κ*B) and leads to the enhancement of the transcriptional activation of iNOS and more cell damage. These mechanisms are partially inhibited by the NMDA receptor antagonist and may be used as an appropriate therapeutic target [8, 9].

**Figure 1:**
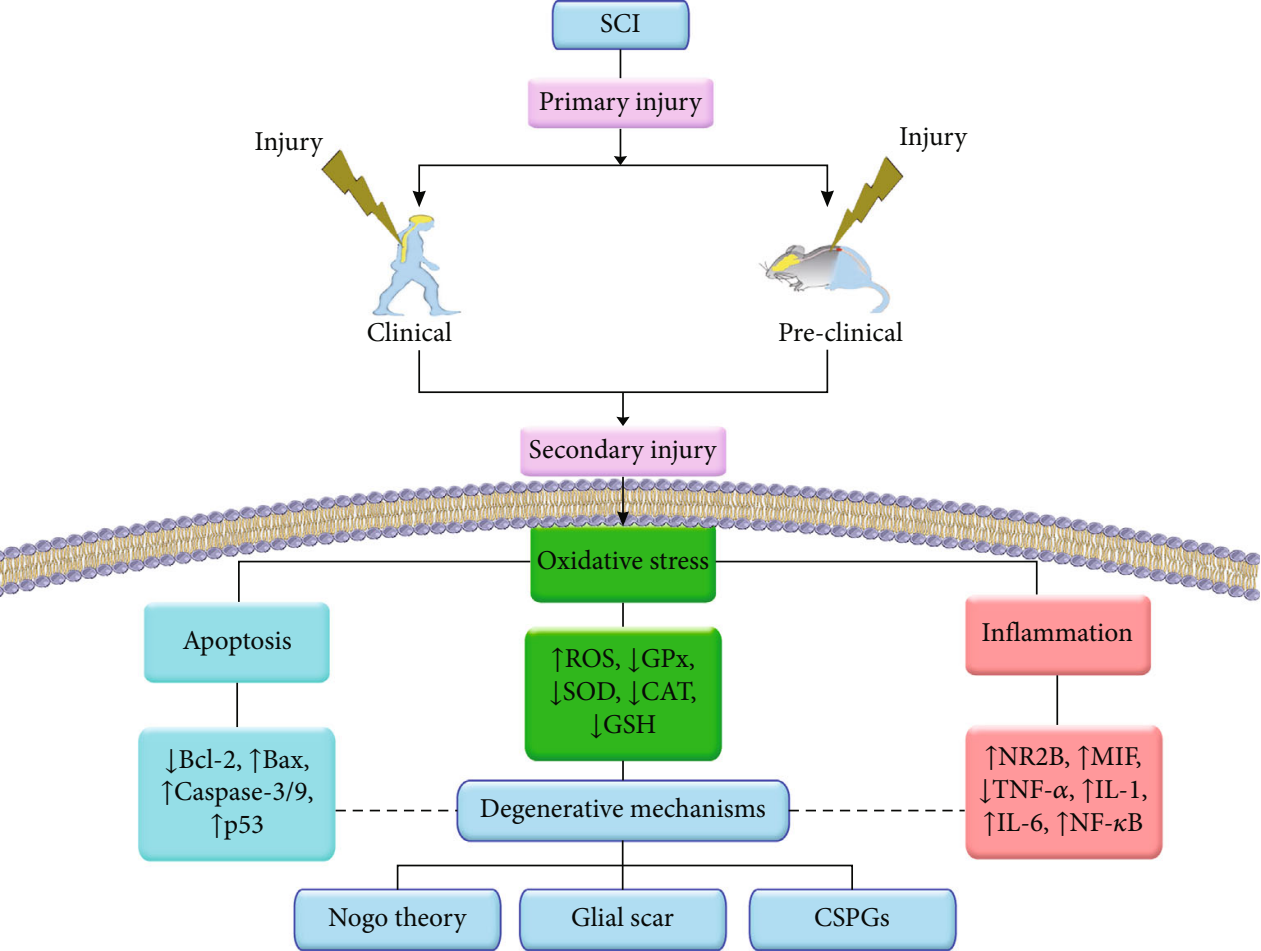
The pathophysiology and proposed targets of SCI. CAT: catalase; CSPGs: chondroitin sulfate proteoglycans; GSH: glutathione; GPx: glutathione peroxidase; IL: interleukin; MIF: macrophage migration inhibitory factor; NF-*κ*B: nuclear factor-*κ*B; NR2B: N-methyl-D-aspartate receptor subtype 2B; ROS: reactive oxygen species; SCI: spinal cord injury; SOD: superoxide dismutase; TNF-*α*: tumor necrosis factor-alpha.

Altogether, it seems that oxidative stress, inflammation, and apoptosis play key destructive roles in the pathophysiology of SCI.  Figure 1 shows the critical role of inflammation, oxidative stress, and interconnected pathways during SCI (Figure 1).

## 3. The Pivotal Role of Oxidative Stress after SCI

Oxidative stress seems to play a more important role than other factors and mechanisms involved in SCI's pathophysiological processes. Several studies have investigated oxidative stress parameters and antioxidant markers in SCI models. Compression, contusion, transection, hemisection, and ischemia-reperfusion are among the most common models of SCI *in vivo* [25]. The results have shown dysregulated levels of advanced oxidation protein products (AOPP), MDA, GSH peroxidase (GPx), SOD, and catalase (CAT) following SCI [26, 27]. Furthermore, the production of superoxide anion )O_2_^¯^(, H_2_O_2_, and other oxidative derivatives such as nitrogen dioxide, peroxynitrite, carbonate, and ^·^OH is increased [28]. Oxidative stress increases the level of NO, and the production of oxidized protein plays an important role in the pathogenesis of SCI. The produced NO leads to cytotoxic effects, vessel dilation, neuronal apoptosis, neurodysfunction, and neurodegeneration. Enhanced intracellular calcium, increasing Glu, and mitochondrial ROS production can activate the posttraumatic cell death cascade and other harmful mechanisms in the SCI [26, 27]. Besides, oxidative stress exacerbates or initiates various inflammatory and cellular apoptotic pathways, so inhibition of oxidative pathways can significantly reduce the severity of post-SCI. It could be considered one of the most appropriate strategies to treat or prevent the progression of SCI [26]. One of the most substantial cellular defense mechanisms versus xenobiotic damage and oxidative stress is the Keap1-nuclear factor erythroid 2-related factor 2 (Nrf2)/ARE pathway. Attenuating this signaling pathway is one of the best ways to control and completely enclose oxidative stress as a suitable target for preventing and treating related disorders and conditions such as neurodegeneration, malignancy, and cardiovascular and inflammatory diseases [29]. ARE is a *cis*-regulatory element or enhancer sequence that can be found in the promoter region of variant genes encoding cytoprotective proteins and detoxification enzymes. In this line, the transcriptional regulation of ARE-dependent expression proteins and enzymes can be induced and controlled by activating Nrf2 signaling. The Keap1 is an inhibitor of Nrf2, and oxidative stress dissociates Nrf2 from Keap1 and activates the antioxidant genes via binding to the ARE [29].

Many of the studies showed appropriate and acceptable effects in controlling injuries after SCI and exerted their effects through the aforementioned pathway, which shows the great importance of oxidative stress in injuries caused following SCI [30]. Also, activating the Keap1-Nrf2/ARE pathway mentioned as the main mechanism of the neuroprotective effect of sulforaphane could increase the levels of Glu-cysteine ligase and Nrf2 and GSH and diminish the levels of inflammatory cytokines, TNF-*α* and IL-1*β* [31]. In addition, other molecular mechanisms involved in the oxidative stress process have been targeted by active compounds and agents capable of suppressing oxidative stress. Enhancing the activity and the levels of CAT, SOD, glutathione reductase (GR), GPx, MDA, phosphoinositol, GSH, and nicotinamide adenine dinucleotide (NADH), as well as suppressing the expression of ROS, iNOS, phospholipase C gamma (PLC*γ*), nicotinamide adenine dinucleotide phosphate- (NADPH-) dependent LPO, and XO, could be targeted by phytocompounds. Besides, decreasing the level of myeloperoxidase, restoring the membrane potential of mitochondria, enhancing the activity of the phosphoinositide 3-kinase (PI3K)/Akt signaling pathway, and increasing the levels of thioredoxin- (TRX-) 1/TRX-2 mRNA are some of the mechanisms that have been targeted by other bioactive compounds in modulating oxidative stress [9, 32–37].

The involvement of oxidative stress and ROS in the pathogenesis of SCI leads to extensive neuroprotective effects of variant compounds with antioxidative activities in different SCI models. Some of these antioxidants may slow the progression and development of spinal cord damage, and antioxidant-based strategies are considered a suitable approach for SCI treatment.

## 4. Related Interconnection between Oxidative Stress and Inflammatory/Apoptotic Pathways after SCI

As mentioned earlier, oxidative stress, inflammatory reactions, and apoptosis are the leading causes of neuronal damage after SCI, which induce their harmful effects through different mediators. Although the signaling pathways of these factors are somewhat different, they are interconnected to each other, like interconnected links in a chain, and exacerbate each other's effects. Accurate identification of the connections and mechanisms between these factors after SCI can be helpful in the treatment or prevention of SCI.

ROS are critical mediators of oxidative stress pathways and play a significant role in the onset and progression of inflammatory responses. Accordingly, increased ROS generation in the injury site leads to endothelial dysfunction, inflammation, and tissue injury. The oxidative stress-mediated inflammatory response leads to the opening of interendothelial junctions and increases the migration and penetration of inflammatory cells across the endothelial barrier. Besides, overexpression of the antioxidant enzyme such as SOD diminishes adhesion molecules' expression and reduces the leukocyte endothelium binding at the site of inflammation [38, 39].

On the other hand, the progression of oxidative stress leads to DNA breaks and mitochondrial damage, followed by a reduction in the alteration of membrane permeability and transmembrane potential, facilitating the release and production of apoptotic factors. It is documented that ROS could play both the inhibitory and activating effects in NF-*κ*B signaling [38, 40]. The lower level of ROS leads to the induction of cell survival responses, while the apoptosis and death processes activate in higher doses of ROS [41]. Inactivating the phosphorylation of IkB*α*, as an inhibitory protein, is considered the main transcription factor of ROS, while H_2_O_2_ has an impact on the phosphorylation of IkB*α*, which leads to IkB*α* degradation and NF-*κ*B activation. Accordingly, the NF-*κ*B signaling pathway affects variant antioxidant proteins such as CAT, SOD, TRX-1, and TRX-2. In this line, activating the transcription of main enzymes involved in ROS production, such as inducible NO synthase, NADPH oxidase, cyclooxygenase (COX), and lipoxygenases, affects the intracellular levels of ROS [38, 40].

Furthermore, oxidation protein products in the Sprague Dawley rat model of SCI could affect the microglia-mediated neuroinflammation through the mitogen-activated protein kinase (MAPK)/NF-*κ*B signaling pathway, activating NADPH oxidase and causing pyroptosis. The process could pass through expressing proinflammatory cytokines and cleavage of Gasdermin-D (GSDMD) during the secondary phase of SCI [42]. Besides, the role of NADPH oxidase and myeloperoxidase generated by immune cells in damaged areas contributes to oxidative damage and inflammation.

As mentioned earlier, mechanical injury to the cord tissue leads to damage to blood vessels. It causes sequestration and penetration of inflammatory molecules such as TNF-*α* and other inflammatory molecules to the affected area. Besides, neuronal damage causes the release of Glu into the extracellular space, which leads to the activation of cytotoxic cascades and an increase in glial and neuronal calcium, ultimately increasing neuronal death. Glu activates microglia and enhances the release of inflammatory molecules, like CD95 ligand and TNF-*α* that, combined with Glu, facilitate necrotic cell death and activate apoptotic pathways [22, 43]. As studies have shown, inflammatory responses, like oxidative stress responses, lead to neuronal apoptosis and neuronal damage. It seems that anti-inflammatory drugs may be a viable option to prevent the progression of nerve cell damage after SCI [43].

From the apoptotic point of view, ROS activate the tumor suppressor protein p53 in severe stress. p53, in turn, downregulates the prosurvival proteins like survivin, B cell lymphoma 2 (Bcl-2), Bcl-XL, and IAPs and upregulates the proapoptotic proteins that actuate the regulation of the apoptotic transcriptional profile. Also, the transcription of proapoptotic genes such as Bid, Bax, p53 upregulated modulator of apoptosis (PUMA), apoptotic protease activating factor 1 (Apaf-1), and Noxa is activated by p53. Cytosolic p53 penetrates the mitochondria and directly interacts with antiapoptotic and proapoptotic proteins (e.g., Bcl-2, Bcl-XL, Mcl-1, Bax, and Bak, respectively), facilitating apoptosis and releasing proapoptotic factors. Direct activation of the Bax through its structural rearrangement and increasing the mitochondrial membrane permeabilization are other mechanisms of p53. In addition, DR-4, DR-5, Fas, and FasL are some of the other extrinsic proapoptotic factors that are altered by p53 [41, 44] (Figure 1).

## 5. Secondary Metabolites in Combating Oxidative Stress after SCI

Plant secondary metabolites are constructed by plants and considered important classes of natural products that can show significant direct or indirect roles in the normal growth, reproduction, and development of the organism. Furthermore, plant secondary metabolites have undeniable and vital effects on the plant defense system against environmental stresses and pathogenic attacks [45]. It was documented that these compounds have shown remarkable biological activities such as antioxidant, anticancer, anti-inflammatory, antimutagenic, immunostimulatory, antibacterial, antiapoptosis, and neuroprotective effects [46–48]. Phenolic compounds, alkaloids, terpenoids, and sulfur-containing compounds are the most known classes of secondary metabolites that express their neuroprotective effects through antioxidant and anti-inflammatory effects [47–49].

As mentioned earlier, one of the most influential factors after SCI is oxidative stress, inflammation, or apoptosis, and secondary metabolites affect and inhibit these pathways through various mechanisms. Suppressing the expression, activity, and levels of MDA, matrix metallopeptidase- (MMP-) 9, TNF-*α*, COX-2, IL-1*β*, IL-6, NADPH oxidase, thiobarbituric acid reactive substance (TBARS), myeloperoxidase (MPO), eNOS, nitrotyrosine, and poly(ADP-ribose) polymerase (PARP) formation and enhancing the levels of SOD, CAT, GPx, and GSH are the main antioxidant, anti-inflammatory, and antiapoptotic mechanisms mentioned for secondary metabolites after SCI. Also, it has been proved that these compounds apply the aforementioned alterations by interfering with the PI3K/Akt/mammalian target of rapamycin (mTOR), Akt/Nrf2, extracellular signal-regulated kinase (ERK) 1/2, Janus kinase- (JAK-) signal transducer and activator of transcription (STAT), and Keap1-Nrf2/heme oxygenase-1 (HO-1). Additionally, catalytic/modifying subunit of glutamate-cysteine ligase (GCLc/GCLc), p38MAPK, transforming growth factor-beta (TGF-*β*)/Smad, MAPK/JNK/NF-*κ*B, sirtuin 1 (SIRT1)/peroxisome proliferator-activated receptor gamma coactivator 1-alpha (PGC-1*α*), ERK/NADPH oxidase, and toll-like receptor-4 (TLR4) signaling pathways are modulated by phytochemicals [9, 50–54].

## 6. Polyphenols Target Upstream Mediators of Oxidative Stress after SCI

Different natural polyphenolic/phenolic compounds have been used to alleviate oxidative stress after SCI [55]. These compounds modulate several dysregulated pathways/mediators, including preventing the formation of ^·^OH and inhibiting the repair of molecules after a free radical attack. Such phytochemicals have previously been shown as potential neuroprotective agents in other neurodegenerative conditions associated with oxidative stress [56, 57].

### 6.1. Quercetin

**Figure 2:**
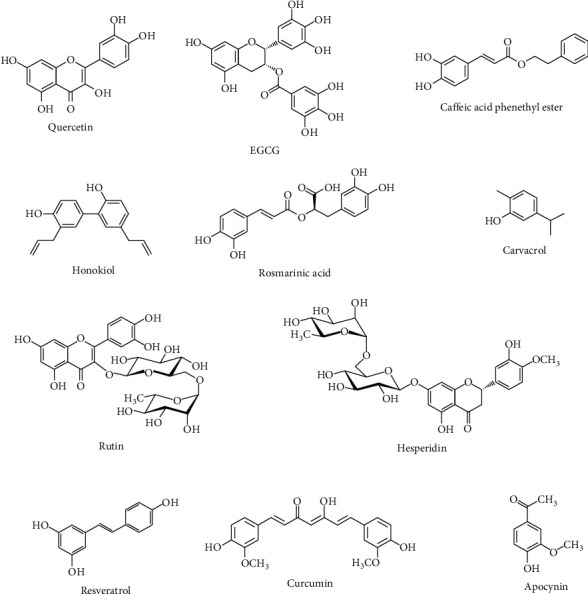
Selected chemical structures of polyphenols.

Quercetin (C_15_H_10_O_7_) (Figure 2) is a flavonoid extracted from various vegetables, fruits, and grains. It has shown beneficial biological properties, including anti-inflammatory, anticarcinogenic, antioxidant, and antiviral effects. Quercetin has also shown the ability to inhibit lipid peroxidation and capillary permeability to stimulate mitochondrial biogenesis in ameliorating neuronal dysregulation and mental/physical dysfunction [58, 59]. Quercetin can directly scavenge ^·^OH, superoxide anions, and LPO via its phenolic hydroxyl groups [60]. In addition, quercetin can bind transition metals and attenuate oxidation and reduction to form metal chelates to inactivate transition metals, such as copper and iron [61]. The neuroprotective effects of quercetin have been extensively demonstrated in several animal models. It significantly suppressed GSH levels and MPO activity following brain trauma [62]. Quercetin also increased the activity of GPx, SOD, and CAT in traumatic brain injury [63], decreased the elevated MMP-9 level [64], and also activated the brain-derived neurotrophic factor (BDNF), tropomyosin receptor kinase B (TrkB), and PI3K/Akt signaling pathway in cerebral focal ischemia [65]. Additionally, quercetin (30 mg/kg, i.p.) inhibited oxidative stress, spinal production of cytokine, and glial cell activation of glial fibrillary acidic protein (GFAP) and ionized calcium-binding adaptor protein 1 (Iba1) mRNA expression [66]. In another study, i.p. administration of the quercetin in the dose of 20 mg/kg/day for 10 days reduced monosodium Glu-induced neurotoxicity in spinal motor neurons by inhibiting p38MAPK, exerting anti-inflammatory effects, antagonizing oxidative stress, and enhancing GFAP expression [67]. Azevedo et al. showed that quercetin (25, 50, and 100 mg/kg, i.p.) inhibited oxidative stress-induced damage in dorsal horn neurons by suppressing LPO [68], which was in line with the results of Liu et al. [69]. Sustained-release poly(lactic-co-glycolic acid)- (PLGA-) polyethylene glycol- (PEG-) PLGA hydrogel formulation of quercetin (50 and 100 mg/mL) reduced oxidative damage and inflammation in the spinal cord and also promoted nerve cell survival, nerve regeneration, and motor function recovery in rats with brachial plexus avulsion [70]. Following SCI, i.p. injection of 20 mg/kg of quercetin for 7 days targets BDNF and JAK2/STAT3 signaling pathways [71] and regulated the secondary oxidative stress by inhibiting the p38MAPK/iNOS signaling pathway [61]. Schültke et al. reported that administration of quercetin in a dose of 0.25 *μ*mol/kg led to a lower MPO expression in damaged spinal cord tissue [72]. In addition, single-dose quercetin within the first 72 hours after SCI enhanced total antioxidant levels and decreased the levels of NO and MDA [73]. In this regard, quercetin also elevated total antioxidant capacity and paraoxonase activity after SCI in rats [74]. In another study, the administration of 20 mg/kg quercetin showed advantageous effects against SCI-induced oxidative damage via its antioxidant and anti-inflammatory effects [75]. In a rat model of SCI, Wang et al. reported that quercetin (50 *μ*mol/kg) combined with human umbilical cord mesenchymal stromal cells reduced proinflammatory cytokines (e.g., IL-1*β* and IL-6) while increased anti-inflammatory cytokines (e.g., IL-4 and IL-10), which are in a near interconnection with oxidative pathways. The treatment also improved macrophage polarization, neurological functional recovery, and axonal preservation and decreased the cystic cavity size [76]. An *in vivo* and *in vitro* study revealed that quercetin (7.5 mg/kg, i.p.) inhibited necroptosis of oligodendrocytes via suppression of the STAT1 and NF-*κ*B pathway after SCI [77]. Jiang et al. reported that 100 mg/kg of quercetin remarkably decreased ROS production, TNF-*α*, IL-1*β*, and IL-18 after SCI in female rats [78].

Overall, quercetin seems to be a hopeful agent in modulating oxidative stress following neuronal damage and SCI.

### 6.2. Epigallocatechin Gallate

Epigallocatechin gallate (C_22_H_18_O_1_) (EGCG) or epicatechin is the main compound of tea catechins. Most of the biological activities of green tea extracts are linked to this composition [79]. Several experimental studies have reported that EGCG causes neuroprotection against neurodegenerative diseases [80], brain injury [81], SCI [82], and peripheral nerve damage [83] through its antiapoptotic, anti-inflammatory, and antioxidant properties [84]. The presence of hydroxyl groups in rings B and D of the catechins makes them bind free radicals [85]. Different concentrations (50–200 *μ*g/mL) of green tea polyphenols for 24 hours protected spinal neurons against oxidative stress caused by H_2_O_2_ [86]. An *in vitro* study displayed that treating PC12 cells with 0, 125, 250, 500, 1000, and 2000 *μ*mol/L concentrations of EGCG reduced ROS production through the SIRT1/PGC-1*α* signaling pathway [87]. Different doses (10, 25, or 50 mg/kg, i.p.) of EGCG also attenuated NADPH/neuronal nitric oxide synthase (nNOS) expression after peripheral nerve injury [83] and inhibited neurotoxicity through activation of the cAMP response element-binding protein (CREB)/BDNF/TrkB and PI3K/Akt signaling pathway in doses of 25, 50, or 75 mg/kg for 18 days in mice [88]. A 50 mg/kg dose of EGCG protected the rat spinal cord from secondary damage by decreasing MPO activity, iNOS, TNF-*α*, IL-1*β*, COX-2, and PARP expression [89]. Khalatbary et al. also found that i.p. injection of EGCG in the dose of 50 mg/kg, immediately and 1 h after SCI, reduced MDA [90]. Consequently, an *in vitro* study indicated that EGCG at a concentration of 5 *μ*M for 48 h reduced oxidative stress levels and protected motor neurons in the organic culture of the rat spinal cord [91]. Coadministration of 17 mg/kg of EGCG and 60 mg/kg of curcumin for 28 days modulated macrophage inflammatory protein 1-alpha (MIP-1*α*), IL-1*β*, IL-4, and IL-6 as cross-talk inflammatory mediators with oxidative stress [92]. Administration of 30 mg/kg of EGCG (i.p.) for a week after SCI reduced thermal hyperalgesia via downregulation of RhoA and TNF-*α* in mice [93]

In this line, 50 mg/kg of protocatechuic acid (i.p.), an essential metabolite of antioxidant polyphenols in green tea, significantly decreased the expression of inflammatory mediators such as TNF-*α*, IL-1*β*, COX-2, iNOS, and MMP-9 in rats after SCI [94]. So, EGCG seems to have a bright future in combating oxidative stress post-SCI.

### 6.3. Caffeic Acid Phenethyl Ester

Caffeic acid phenethyl ester (C_17_H_16_O_4_) or phenylethyl caffeate is a component of honeybee propolis [95]. Its biological activities include antioxidant [96], anti-inflammatory [97], antibacterial [98], anticancer, and cytotoxic properties [99], which is due to related hydroxyl groups in the catechol ring [100]. Caffeic acid phenethyl ester is a potent inhibitor of NF-*κ*B [101] and protein tyrosine kinase [102]. It suppresses lipoxygenase activities [103] and blocks calcium-induced cytochrome c release in the hypoxic-ischemic brain injury models [104]. From another view, caffeic acid phenethyl ester repressed the formation of superoxide anion and XO activity in eukaryotic cells [105] and reduced MPO and Na^+^/K^+^ ATPase activity during ischemia-reperfusion injury [106]. Caffeic acid phenethyl ester activated the expression of HO-1 through Nrf2 activation associated with the ERK signaling pathway [107]. It binds to Keap1 and disrupts the Nrf2/Keap1 complex, thereby enhancing Nrf2 binding to ARE [108]. Intrathecal injection of 1 *μ*g/kg caffeic acid phenethyl ester after SCI led to a decrease in MDA, LPO, and total oxidant activity. It increased antioxidative agents [109], while decreasing tissue and serum levels of IL-6 post-SCI [110]. In this line, Ak et al. confirmed the effects of i.p. injection of caffeic acid phenethyl ester (10 *μ*g/kg) in reducing the levels of TNF-*α* and IL-1*β* post-SCI [111]. Administration of 10 *μ*mol/kg (i.p.) of this phytochemical improved motor function and reduced lesion size by decreasing the expression of IL-1*β*, NOS, and COX-2 following SCI [112]. Administrating 10 *μ*mol/kg (i.p.) of caffeic acid phenethyl ester before surgery diminished ischemic injury in the spinal cord through scavenging free radicals and providing a better microcirculatory environment by preventing endothelial cell lysis through proteases from activated leukocytes [113]. It also exerted neuroinflammatory effects via inhibiting ROS and catalytic activity of iNOS in the concentration of 50 *μ*mol/mL [114].

### 6.4. Honokiol

Honokiol (C_18_H_18_O_2_) is a pleiotropic lignan presented in *Magnolia grandiflora* [115]. It is known for its therapeutic properties such as antioxidant [116], anti-inflammatory [117], analgesic [118], antidepressant [119], antitumorigenic [120], and neuroprotective [121] effects. Several studies showed that honokiol could decrease oxidative stress factors in various organs such as the heart [122], liver [123], kidney [124], and brain [125]. Honokiol inhibited ROS production via the ERK/NADPH oxidase pathway in microglial cells [126]. It also activated Nrf2 [127], inhibited XO [128], and modulated the PI3K/Akt pathway [117] to exert neuroprotective responses. Besides, honokiol preserved mitochondrial respiratory chain enzyme, inhibited ERK/protein kinase C (PKC) pathways [129], and modulated lipopolysaccharide- (LPS-) induced NO expression by targeting PKC, MAPKs, and NF-*κ*B [130]. In a rat model of SCI, 20 mg/kg of honokiol (i.p.) reduced the production of proinflammatory cytokines and inhibited neutrophil infiltration and microglial activation, which are all in a near link with oxidative mediators [131]. In the ischemic brains, 10 *μ*g/kg of honokiol (i.p., twice) maintained Na^+^/K^+^-ATPase activity and mitochondrial function against oxidative stress [121] and inhibited neutrophil infiltration and ROS production [132].

### 6.5. Rosmarinic Acid

Rosemary (C_18_H_16_O_8_), with a molecular formula of C_18_H_16_O_8_, contains several bioactive polyphenol components, including carnosic acid, carnosol, genkwanin, rosmarinic acid, and rosmanol [133]. Rosmarinic acid is an ester produced of caffeic acid that was first found in *Rosmarinus officinalis*. Rosmarinic acid has remarkable biological effects, including anticancer, antiviral, antibacterial, antiaging, antioxidant, antidiabetic, hepatoprotective, nephroprotective, cardioprotective, antiallergic, antidepressant, and anti-inflammatory activities [134]. The antioxidant activity of rosmarinic acid, along with its effects on signaling pathways and gene expression, contributes to most of its biological properties [135]. Rosmarinic acid decreased LPO and elevated GSH levels in HepG2 hepatoma cells [136]. Besides, it reduced p38MAPK, p-JNK, NF-*κ*B, IL-1*β*, IL-6, and TNF-*α* but increased activity of CAT, SOD, glutathione S-transferase (GST), and GPx in diabetic rats [137]. Moreover, it inhibited ROS-dependent MMP-2 activity by targeting the Nrf2 antioxidant system in hepatic cells [138], increased SOD, CAT, HO-1, and Nrf2 activity, and was significantly decreased by UVB radiation [134]. Rosamrinic acid (0.5, 2.5, 5, and 10 *μ*g/mL) repressed oxidative stress in C6 glial cells by inhibiting LPO and reducing COX-2 expression and iNOS [139]. Rosmarinic acid significantly attenuated the levels of TNF-*α*, iNOS, apoptotic factors, Iba1, TLR4, and GFAP after chronic contraction injury in rats [140]. Treatment with rosmarinic acid at the doses of 10, 20, and 40 mg/kg (i.p.) for 14 days suppressed the spinal glial cell activation. It inhibited the expression of inflammatory markers, as well as activating AMPK in peripheral nerves and dorsal root ganglia, which might also have contributed to its neuroprotective actions [141]. An *in vitro* study in PC12 cells showed that the antioxidant effects of 10 mM rosmarinic acid are mediated by the Akt/glycogen synthase kinase-3*β* (GSK-3*β*) pathway and increasing Nrf2 activity [142].

### 6.6. Carvacrol

Carvacrol (C_10_H_14_O) or cymophenol is a phenolic monoterpenoid derivative from cymene. Carvacrol has shown various pharmacological activities such as anxiolytic [143], antidepressant [144], antibacterial, antioxidant [145], antitumor, antigenotoxic, antimutagenic [146], analgesic, anti-inflammatory [147], hepatoprotective, and antihepatotoxic activities [148]. Carvacrol protects the brain, liver, and kidney against oxidative stress and significantly increases GSH, SOD, CAT, and GPx levels [149]. Additionally, carvacrol enhanced the expressions of Nrf2 and ERK1 inhibited by cadmium in PC12 cells [150]. It also made cytoprotective effects through HO-1 in cells exposed to H_2_O_2_ [151] and inhibited the MAPK/JNK-NF-*κ*B signaling pathway in cells exposed to iron ions [152]. The overload of iron ions leads to a reaction with oxygen and the production of oxidative damage, including mitochondrial dysfunction and LPO via Fenton reaction [153]. Carvacrol also exhibited modulatory effects on the expression of pro- and anti-inflammatory cytokines [154]. It has also suppressed transient receptor potential ankyrin 1 (TRPA1) receptors and decreased the production/release of proinflammatory cytokines and markers like TNF-*α*, IL-1*β*, MPO, NF-*κ*B, and COX-2, as well as oxidative stress factors, including MDA, GSH, and NO levels following i.p. administration (25, 75, and 150 mg/kg) [155]. However, more experiments are needed toward a better future of the neuroprotective effects of carvacrol after SCI via suppressing oxidative pathways.

### 6.7. Rutin

Rutin (C_27_H_30_O_16_), also known as rutoside or vitamin P (C_27_H_30_O_16_), is a flavonol glycoside obtained from buckwheat [156]. Rutin is known for various pharmacological activities, including cytoprotective, antioxidant [157], anticancer [158], vasoprotection [159], neuroprotection [160], and post-SCI neuroinflammation [161] activities. Rutin modulated the MAPK [162] and iNOS/Nrf2 signaling pathway [163] and reduced oxidative stress by an enhancement in CAT activity along with a decline in LPO and protein carbonyl content [164]. Rutin also attenuated ischemic neural apoptosis by inhibiting LPO and p53 expression, an increment in antioxidant defense enzymes [161], and a decrement in ROS production [165]. It improved diabetic neuropathy by reducing oxidative stress through HO-1 and Nrf2 in rats [166]. Employing 100 mg/kg of rutin also upregulated BDNF, CREB, and ERK1 gene expression in the hippocampus [167] and protected PC12 cells exposed with sodium nitroprusside via modulating the PI3K/Akt/mTOR and ERK1/2 pathway [168]. Oral administration of 10 mg/kg of rutin for 3 weeks inhibited oxidative stress and inflammation by targeting the NOS-mediated NF-*κ*B signaling pathway [169]. Besides, Zhang and Ma showed that 10 *μ*mol/kg of rutin (i.p.) reduced the expression levels of MIP-2 and p-Akt in a rat model of SCI [170]. Also, rutin (30 mg/kg) decreased oxidative stress-related markers and inflammatory cytokines (e.g., IL-1*β*, IL-6, and TNF-*α*) by suppressing the p38MAPK pathway after SCI [171]. Another study showed that i.p. injection of rutin (50 and 100 mg/kg) for 3 days significantly attenuated the levels of ROS, MDA, IL-1*β*, IL-18, and TNF-*α* [161]. In an *in vitro* study, rutin protected cells from H_2_O_2_-induced oxidative stress and apoptosis through regulation of ROS, suppression of LPO, protection of the intracellular antioxidant system, and regulation of the Bax/Bcl-2 ratio and NF-*κ*B/p65 signaling pathway [172]. Rutin also prevented oxidative DNA damage and neuronal death induced by nutrient deprivation conditions [173]. Also, it was reported that i.p. injection of 30 mg/kg of rutin for 3 days after SCI plus mild hypothermia reduced inflammatory factors through repressing the TGF-*β*/Smad pathway in the SCI model [174].

### 6.8. Hesperidin

Hesperidin (C_28_H_34_O_15_) is a flavanoglycone isolated from citrus fruits that possesses anti-inflammatory, antioxidant [175], anticancer [176, 177], and antiapoptotic [178] activities. Hesperidin attenuated oxidative stress and inflammation via modulation of TGF-*β*1/Smad3, Nrf2/ARE/HO-1, and peroxisome proliferator-activated receptor *γ* (PPAR*γ*) signaling [179]. Additionally, it inhibited LPO in Parkinson's disease [180], increased GSH and total antioxidant capacity, and reduced H_2_O_2_ and MDA levels. Hesperidin inhibited GSK-3*β* activity in Alzheimer's disease [181] and also activated antioxidant enzymes (XO, GST, and GR) [182]. An *in vitro* study based on oxidative stress showed that activation of the ERK/MAPK signaling by hesperidin is involved in the expression of HO-1 and Nrf2 [183]. Hesperidin also upregulated the Keap1-Nrf2/HO-1 pathway *in vitro* [184] and improved the antioxidant defense system via activation of Nrf2/ARE/HO-1 in kidney tissue [185]. Consequently, it suppressed oxidative stress and inflammation through activating Nrf2/HO-1/ARE and PPAR*γ* pathways [179]. In a recent study by Heo et al., hesperidin (100 mg/kg, i.p.) for 14 days (from 7 days prior to SCI to day 7 post-SCI) decreased oxidative stress and inflammation by upregulation of the HO-1/Nrf2 pathway after SCI [186]. At the same dose, hesperidin led to a decrease in TNF-*α*, IL-1*β*, and IL-6 in the spinal cord tissue [187].

### 6.9. Resveratrol

Resveratrol (C_14_H_12_O_3_) is a polyphenol belonging to stilbenoids, possessing two phenol rings and an ethylene group. Resveratrol is present in various plants such as plums, peanuts, mulberries, and grapes [188]. In addition to its antiapoptotic [189], anti-inflammatory, antinociceptive [190], and anticancer [191] properties, the antioxidant potential of resveratrol is of the most important biological effects of resveratrol [192]. Resveratrol scavenges free radicals through antioxidant effects and suppresses the iNOS/p38MAPK pathway [193]. Treatment with 50 *μ*M of resveratrol maintained the spinal cord dorsal column against hypoxic injury through activating Nrf2 [194] and decreasing pancreatic oxidative damage via downregulating NF-*κ*B and PI3K pathways [195]. Besides, resveratrol diminished inflammatory hyperalgesia by targeting antioxidant enzymes, ROS, and ERK activation [196]. It also exerted antioxidant effects following myocardial ischemia through the Nrf2/ARE signaling pathway [197]. In another study by Tamaki et al., oral administration of resveratrol in the dose of 10 mg/kg enhanced antioxidant gene expression by activating SIRT1/AMPK and Nrf2 pathways [198]. Resveratrol (5 mg/kg orally for 30 days) protected against oxidative stress through Nrf2/Keap1 signaling [199]. An *in vitro* study exhibited that resveratrol protected astrocytes from oxidative damage by inhibiting ROS and NF-*κ*B, while upregulating Nrf2 expression. They reported that resveratrol was more effective in late hours after oxidative trauma [200]. Resveratrol in the dose of 10 mg/kg protected the spinal cord from spinal ischemia-reperfusion injury via increasing NO release and decreasing oxidative stress [74]. Ates et al. showed that administration of resveratrol (100 mg/kg, i.p.) and methylprednisolone (30 mg/kg, i.p.) immediately after trauma led to a decrease in MDA, XO, and NO activity and an increase in the GSH level following SCI [201]. Also, in another study, the same dose of resveratrol was associated with the regulation of SIRT1 expression and the suppression of NF-*κ*B activity in lung tissue after SCI [202]. A meta-analysis reported that resveratrol significantly improved motor function after SCI and also elevated SOD levels while diminished MDA levels following SCI [203]. Resveratrol administration (7 and 14 mg/kg) from 4 days before the experiment, until the end of the experiment, resulted in the suppression of proinflammatory cytokines (NF-*κ*B and TNF-*α*) and upregulation of antioxidant mediators, including Nrf2, NAD(P)H:quinone oxidoreductase (NQO1), and HO-1 [204]. As one of the resveratrol derivatives, oxyresveratrol showed twice more antioxidant capacity [205]. Treatment of SCI with oxyresveratrol (10 and 20 mg/kg, for 4 weeks) reduced inflammation and oxidative stress by enhancement of Nrf2, SOD, GPx, and GSH and decrement of MDA, IL-1*β*, IL-6, TNF-*α*, NF-*κ*B, COX-2, and iNOS [206]. Administration of 40 mg/kg (i.p.) polydatin, as a glycoside and precursor form of resveratrol [207], improved SCI by inhibiting oxidative stress via Nrf2/HO-1 signaling [208]. Polydatin in 20 and 40 mg/kg doses (i.p., 30 min after surgery) attenuated SCI complications by reducing microglial inflammation via suppressing the iNOS and NLR family pyrin domain containing 3 (NLRP3) inflammasome pathway [209]. Polydiatin, in combination with bone marrow stem cells (BMSCs), ameliorated SCI by increasing cell survival and reducing oxidative stress through the Nrf2/ARE pathway [210].

In another study, 100 mg/kg of resveratrol (i.p.) exosomes improved motor function by inhibiting apoptosis and activating autophagy through the PI3K signaling pathway [211]. Resveratrol also blocks the Notch signaling pathway to limit SCI [212]. Liu et al. reported that resveratrol was an effective treatment for SCI by reducing neuronal cell apoptosis and inflammation [213]. In a recent study by Song et al., 10 mg/kg of resveratrol and puerarin-loaded nanoparticles attenuated CAT, SOD, and GSH antioxidant levels [214]. Resveratrol protected the spinal cord against ischemia-reperfusion injury via decreasing oxidative stress and NO release [215]. Treatment with 400 mg/kg of resveratrol for 10 days attenuated inflammation and oxidative stress after a rat model of SCI [216]. The i.p. injection of resveratrol (50 and 100 mg/kg) immediately after SCI markedly improved the activity of Na^+^/K^+^-ATPase and reduced the production of MDA in the injured spinal cord tissue [217].

### 6.10. Curcumin

Curcumin (C_21_H_20_O_6_) is a natural phenol extracted from *Curcuma longa*. It has shown several pharmacological effects, such as anticancer [218], antiapoptotic, antioxidant, and anti-inflammatory properties [219]. Unlike other natural antioxidants containing either diketonic or phenolic groups, curcumin has both groups that help scavenge free radicals [220]. Curcumin decreased oxidative stress by activating the Keap1/Nrf2/ARE signaling in diabetic rats [221]. Two other studies confirmed that curcumin decreased MDA levels and increased SOD and GPx levels after spinal cord ischemia-reperfusion injury [222, 223]. Curcumin (300 mg/kg, i.p.) also improved ischemic brain injury by upregulating the Akt/Nrf2 pathway in a rat model of neurotoxicity [224]. It also activated the Nrf2/ARE signaling pathway after traumatic brain injury [225]. It upregulated HO-1 through PI3K/Akt/Nrf2 and reduced HO-2 in SH-SY5Y cells exposed to oxidative stress [226]. In a recent study, Daverey and Agrawal showed that 10 *μ*M of curcumin and riluzole protected astrocytes derived from the human spinal cord against oxidative stress via Nrf2/HO-1 signaling [227]. Previously, Jiang et al. showed that curcumin in different concentrations (5, 10, 15, 20, and 25 mM) for 2 h activated Nrf2 target genes in primary spinal cord astrocytes, attenuated the level of intracellular ROS, and decreased oxidative damage and mitochondrial dysfunction [228]. Additionally, i.v. injection of curcumin (50, 100, and 200 mg/kg) for 7 days decreased p38MAPKs and MAPK kinase phosphorylation levels as key mediators in the microglia-mediated inflammatory response. They also showed that curcumin downregulated the expression levels of NF-*κ*B post-SCI [229]. Curcumin (100 mg/kg) induced the Nrf2 signaling pathway in the SCI model [230] and decreased the levels of NO, ^·^OH, and MDA following SCI [231]. In another study, curcumin (200 mg/kg, i.p.) immediately after the injury increased GPx, SOD, and CAT post-SCI [232]. Curcumin also decreased MDA concentration at the dose of 300 mg/kg [233].

Curcumin protected astrocytes from oxidative stress through the Nrf2/HO-1 signaling pathway [227]. In a dose of 200 mg/kg, for 8 weeks (i.p.), curcumin also decreased the levels of IL-6, IL-8, and TNF-*α*, as well as reduced astrogliosis and ameliorated motor function following SCI [234]. Also, curcumin in the dosage of 30 mg/kg (i.p.) for 7 consecutive days reduced the expression of NOS and NMDA following ischemic SCI [235] and in the dosage of 50 mg/kg (i.p.) significantly reduced the expression of IL-1*β* and NO in SCI mice [236]. Lin et al. showed the neuroprotective effects of curcumin by decreasing the expression of oxidative mediators (e.g., MDA) and iNOS in the SCI model study both *in vitro* (1 *μ*M) and *in vivo* (40 mg/kg, i.p.) [237]. In another study, i.p. injection of curcumin was associated with decreasing the level of proinflammatory cytokines, such as IL-1*β*, TNF-*α*, and NF-*κ*B, as well as inhibition of the generation of TGF-*β*1 and TGF-*β*2 [238]. Treatment with 40 mg/kg of curcumin caused a decrease in inflammatory factors and apoptosis following SCI and LPS-treated astrocytes [239].

Several other studies have shown that curcumin has anti-inflammatory, antiapoptotic, and antioxidant effects after SCI [229, 240–244] (Table 1).

### 6.11. Mangiferin

Mangiferin (C_19_H_18_O_11_) is a bioactive xanthonoid isolated from different parts of the mango. It is a strong antioxidant [245] with numerous health-related properties, including immunomodulatory [246], antiviral [247], anti-inflammatory [248], antidiabetic [249], anticancer [250], and analgesic effects [251]. Mangiferin neutralizes free radicals by forming mangiferin-iron complexes and efficiently suppresses LPO and DNA damage [245]. In an *in vivo* study, 50 *μ*mol/L of mangiferin for 4 h protected mice against cadmium chloride by reducing LPO content and incensement of GSH, CAT, GST, and SOD activity [252]. In an *in vitro* study, mangiferin enhanced the Nrf2 level, modulated NQO1 expression, and induced ROS levels [253]. Also, treatment with 20 *μ*M of mangiferin for 2 h maintained renal cells against oxidative stress by targeting PI3K/Akt and Nrf2 signaling pathways [254]. In addition, in a neuronal injury model, 20 and 100 mg/kg mangiferin (i.p.) activated the Nrf2/HO-1 cascade in a dose-dependent manner [255]. Mangiferin (20 and 100 mg/kg, i.p.) for 30 days effectively reduced the level of MDA while increased SOD and CAT and GPx post-SCI [19]. The neuroprotective effects of mangiferin in doses of 10, 25, and 50 mg/kg for 30 days after SCI were associated with reduced spinal cord edema, inhibition of oxidative stress, inflammatory response, and regulation of Bcl-2 and Bax pathways [256].

### 6.12. Schisandrin B

Schisandrin B (C_23_H_28_O_6_) is a dibenzocyclooctadiene derivative extracted from *Schisandra chinensis* [257]. It has shown promising antioxidant [258], anti-inflammatory [259], hepatoprotection [260], and neuroprotection activities [261]. Schisandrin B prevented cardiotoxicity by reducing oxidative stress, DNA damage, and inflammation by inhibiting the MAPK/p53 pathway [262]. Schisandrin B (50 mg/kg) prevented the progression of liver fibrosis by modulation of Nrf2/ARE and TGF-*β*/Smad signaling pathways [263]. Orally administered Schisandrin B at the dose of 30 or 60 mg/kg alleviated oxidative stress through modulation of the Nrf2/Keap1 antioxidant pathway [264]. The ability of Schisandrin B in activating Nrf2 is related to its biotransformation through cytochrome P450-catalyzed reactions [265]. In another study, a 50 mg/kg dose of Schisandrin B (p.o., for 5 days) diminished oxidative stress and inflammatory responses induced by SCI via inhibition of p53 signaling [266]. Oral administration doses of 20, 40, and 80 mg/kg of Schisandrin B for 5 days before SCI attenuated oxidative stress and inflammation through p38MAPK, ERK1/2, and NF-*κ*B pathways after an ischemia/reperfusion injury model [267].

The antioxidant effects of some other polyphenols/phenolic compounds, including apocynin [268, 269], baicalin [270, 271], gastrodin [272], mangiferin [19], mulberrin [273], salvianolic acid [274, 275], gallic acid [276, 277], kaempferol [278], luteolin [279], naringenin [280], oleuropein [281, 282], pycnogenol [283], silymarin [284], and protocatechuic acid [94], have been shown in Table 1. From the chemical point of view, Figure 2 presents the candidate chemical structures of neuroprotective polyphenols against SCI.

## 7. Extrinsic Molecular Mechanisms of Regeneration Post-SCI: Therapeutic Approaches to Polyphenols

After SCI, axons and dendrites lose their connection to the main neural pathways, and the process is termed Wallerian degeneration and axon searing. In this line, anterograde and retrograde degeneration of neural fibers could be the main factor in the declining tissue mass at the injured spinal cord [292]. One of the goals of SCI-related research is to repair disrupted neural networks and regenerate axons, which is hoped to reconnect descending neural fibers with their original targets [293]. There are several theories of post-SCI regeneration, predominantly the Nogo signaling pathway, the glial scar mechanism, and the chondroitin sulfate proteoglycan-related pathway.

### 7.1. The Nogo Signaling Pathway

In 2000, Nogo-A (reticulon 4) was introduced as the myelin-associated neurite outgrowth inhibitor [294]. It is expressed by oligodendroglia and neurons but not astrocytes [295]. Three Nogo receptors have been reported, NgR1, NgR2, and NgR3 [296]. Many studies reported that blocking Nogo receptors promotes axonal sprouting [297–299]. In an *in vitro* study by Gundimeda et al., polyphenols extracted from green tea prevented antineuritogenic properties of Nogo-A through H_2_O_2_ and laminin receptors [300].

Nogo, myelin-associated glycoprotein (MAG), and oligodendrocyte myelin glycoprotein (OMgp) trigger interconnected receptors that trigger the Ras homolog gene family member A (RhoA) and its effector, Rho-kinase (ROCK), which lead to the inhibition of axonal growth [301]. RhoA controls cellular motility via the regulation of cytoskeletal rearrangements. It also activates phospholipases A2, C, and D and serine/threonine kinases [302], promoting the expression of COX-2 at the transcription level depending on NF-*κ*B [303]. Protein kinase A (PKA) via RhoA phosphorylation regulates the morphological and functional subsequences of cAMP. Besides, RhoA modulates several other cellular functions, such as assembly of extracellular fibronectin, potassium channels, neurotransmitter exocytosis, thrombin-induced cell death, gap junctions, cellular uptake of low-density lipoprotein, expression of cyclin D1, basal muscle tone, IL-8, and neurite growth cones [302]. Reports have shown that SCI upregulates Rho proteins. The Y27632 and fasudil (ROCK-specific inhibitors) significantly reduced IL-6 synthesis, contributing to inflammation-induced CNS regeneration [304, 305].

Phosphatase and tensin homolog (PTEN) catalyzes the conversion of PIP3 to PIP2, followed by Akt activation via phosphoinositide-dependent protein kinase 1 (PDPK1). Akt, in turn, stimulates inflammatory messengers Raf/Rac/NF-*κ*B and thereby inhibits tuberous sclerosis complexes 1 and 2 (TSC1/2) to suppress Ras homolog enriched in brain 1 (Rheb1). Rheb1 activates mTOR to stimulate protein synthesis and cell growth. Activation of Akt also inhibited GSK-3*β*, which disinhibits Wnt/*β*-catenin to stimulate mTOR and cell proliferation. The cAMP turns on phosphokinase A, which inhibits Rho and Rho-kinase. Rho and Rho-kinase inhibit cell growth when activated by axonal growth inhibitors Nogo and CSPG [302]. In line with this, ROS, which increased axonal dieback and degeneration post-SCI, are also needed for axonal regeneration and functional recovery after SCI. ROS via the release of exosomal NADPH oxidase 2 complexes and oxidized PTEN lead to its inactivation, thus stimulating the PI3K/Akt pathway toward axonal regeneration [306, 307].

Some natural substances, such as curcumin, affected Rho expression and reduced the protein levels of phosphokinase C, NF-*κ*B, and RhoA [308]. In another study, curcumin was highlighted as a novel inhibitor of the Rho/ROCK pathway to develop axonal growth following SCI *in vivo*/*in vitro* [309]. As reported on other polyphenols, Álvarez-Pérez et al. showed that EGCG meaningfully downregulated RhoA and astroglial and microglial activity in the spinal cord *in vivo* [93].

### 7.2. The Glial Scar Mechanism

After SCI, astrocytes play a key role in SCI pathology through a phenotypic change called reactive gliosis. Immediately after injury, astrocytes, as a type of glial cells, proliferate and accumulate around the lesion, separating healthy tissue from the surrounding damaged tissue. Reactive astrocytes migrated to the lesion epicenter, and secluded inflammatory cells resulted in tissue repair and functional improvement [310]. Consequently, reactive astrocytes made the astrocytic scar as the main obstacle to axonal regeneration [293]. A number of processes and proteins such as microgliosis [311], nestin [312], neurotrophins [313], albumin [314], oxygen, and glucose deprivation contribute to gliosis [315]. In addition, after SCI, intensive inflammatory response resulted in the activation of resident microglia and facilitated the infiltration of macrophages into the lesion post-SCI. Macrophages play main roles in the inflammation process and are known as proinflammatory (M1) and anti-inflammatory (M2) mediators [316, 317]. Interventions that relieve inflammation, reduce secondary damage after SCI, and modulate the phenotypes of infiltrating macrophages can be a therapeutic strategy to improve functional recovery post-SCI [318]. Microglia also secrete a set of signaling molecules, such as chemokines, cytokines, and growth factors. Many of these factors act via receptors on oligodendrocyte progenitor cells. For example, TNF-*α*, secreted by microglia/macrophages as well as astrocytes in mouse cuprizone-induced lesions, induced the proliferation and remyelination of oligodendrocyte precursor cells (OPCs) via TNFR2 on polydendrocytes [319]. Type I interferon, bone morphogenic proteins [302], and STAT3 knockout inhibited reactive astrocytosis [320]. Blocking STAT3 was associated with preventing astrogliosis, leading to a higher level of inflammation, an increase in lesion volume, and a reduction in motor recovery after SCI. Besides, TGF-*β*1 plays a critical role in regulating glial activation. Proinflammatory cytokines trigger the astrocytes to secrete chemokines that are antagonized by TGF-*β*1 [321].

These findings revealed that curcumin developed axon/neuron protection of the spinal cord through suppressing the formation of glial scars [241] and the inflammatory astrocyte marker (Iba1) [236]. Nanoformulation of curcumin also mitigated glial scar formation and protected the white matter following SCI in rats [322]. By decreasing the formation of glial scars, curcumin and EGCG increased axonal sprouting and modulated MIP-1-alpha, IL-1*β*, IL-4, and IL-6 in rats post-SCI [92]. The synergistic effects of curcumin with mesenchymal stem cells prevented axonal sprouting, glial scar formation, and inflammatory responses in rats following SCI [323]. As glial cells closely connect with NF-*κ*B, curcumin also regulated glial scar formation by inhibiting NF-*κ*B and TGF-*β*-SOX9 [324].

Resveratrol has also shown promising inhibitory effects on NF-*κ*B following SCI [325]. Another study by Zhang et al. showed that resveratrol improves neuronal regeneration of the spinal cord passing through the attenuation of Notch signaling [212]. As another phenolic compound, quercetin provided functional recovery, axonal regeneration, and astrocyte activation in rats post-SCI through BDNF and JAK2/STAT3 signaling pathways [71]. Besides, treatment with quercetin regulated oligodendrocyte necroptosis by inhibiting M1 macrophage/microglial polarization and reducing myelin/axon loss following SCI in rats [77].

Naringenin promoted the remyelination of the spinal cord by regulating the differentiation of oligodendrocyte precursor cells passing through the GSK-3*β*/*β*-catenin pathway [326]. Consequently, triggering axonal sprouting and positive modulation of glial scars, as well as inflammation (e.g., NF-*κ*B) suppression, also resulted from treating with EGCG in rats following SCI [82].

In a previous study reported by Subbarayan et al., caffeic acid hydrogel, another phenolic compound, could improve functional recovery and remyelination after hemitransection SCI. They found that such hydrogel decreased IL-6, TNF-*α*, and neutrophil release. Caffeic acid hydrogel also modulated gamma-aminobutyric acid and glutamine levels, as well as regulation of genes/proteins involved in locomotor activity after SCI [327].  Figure 1 presents the cross-talk between extrinsic dysregulated pathways and intrinsic ones in the pathogenesis of SCI.

### 7.3. Chondroitin Sulfate Proteoglycan-Related Pathways

Studies show that chondroitin sulfate proteoglycans (CSPGs) repel regenerative axons and inhibit oligodendrocyte remyelination/maturation [328]. Destruction of CSPGs by chondroitinase ABC (ChABC) is a potential therapeutic strategy for breaking the inhibitory barrier, synaptic reorganization, and functional recovery of SCI [329]. ChABC may act through multiple mechanisms. Several studies have shown that ChABC degrades chondroitin sulfate proteoglycans and promotes morphological plasticity. It also reduces glial scars and axon dieback, regenerates spinal respiratory pathways, promotes axonal conduction and functional recovery, stimulates remyelination, and enhances migration of oligodendroglial and neural progenitor cell migration [302]. ChABC in combination with neural stem/progenitor cells (NSPCs) improved functional recovery after SCI [330].

Many studies have reported that injured spinal axons will grow across SCI sites transplanted with neural stem cells [331], Schwann cells [332], mesenchymal stem cells [333], olfactory ensheathing glia [334], or BMSCs [335]. The spinal cord has been shown to be regenerated after treatment with Schwann cells [336] or PTEN deletion [337].  Figure 1 also reveals the interconnections between degenerative/regenerative extrinsic mechanisms and oxidative stress post-SCI.

Table 2 indicates the critical role of polyphenols in modulating extrinsic regenerative/degenerative mechanisms following SCI.

## 8. Combination Therapy of Polyphenols and Stem Cells Post-SCI

Stem cells are undifferentiated cells with the potential to differentiate into different cells. In recent years, stem cell therapy for neurodegenerative diseases has shown a promising prospect. Many studies have reported that polyphenols can target and promote the potential of stem cells against neurodegeneration [338]. For example, curcumin induced neuronal differentiation in neural stem cells by activating the Wnt pathway and upregulating the genes required for cell differentiation [339]. Ruzicka et al. reported that combination therapy of curcumin in a high dose (60 mg/kg) and mesenchymal stem cells synergistically improved recovery from the balloon compression-SCI model and modulated expression of ILs, TNF-*α*, and MIP-1*α* [323]. Previously, these results were shown by Ormond et al. [340]. Wanjiang et al. suggested that curcumin in a dose of 100 mg/kg suppressed the apoptosis of human umbilical cord-derived mesenchymal stem cells (hUC-MSCs) through the ERK1/2 pathway. Additionally, a combined administration of curcumin with hUC-MSCs improved motor function in female rats after compression-SCI in a dose-dependent manner [341]. It has also been shown that intrathecal administration of curcumin can improve the functional recovery post-SCI through the spinal cord-NSPCs (SC-NSPCs) by increasing the expression of SC-NSPCs and decreasing the activity of reactive astrogliosis (GFAP) and lesion cavities [342]. In another study, combined therapy of polymer-curcumin with ependymal progenitor/stem cells (epSPCs) by intrathecal injection (10 *μ*M) enhanced functional recovery and axonal growth post-SCI in rats [309]. In addition, concomitant use of human-induced neural and mesenchymal stem cells (iPSC-NSC) with a polyacetal-curcumin nanoconjugate provided neuroprotective and immunomodulatory effects that led to the prevention of axonal degeneration and neuronal death, reduced the injury area, and inhibited the formation of glial scars in chronic stages in a rat model of contusive SCI [343].

In an *in vitro* study, to mimic the microenvironment around transplanted cells in the damaged spinal cord, as a glucoside of resveratrol, polydatin protected BMSCs against oxidative injury caused by H_2_O_2_ through the Nrf2/ARE pathway [210]. Combining BMSCs and a 400 mg/kg dose of green tea polyphenols, for 30 days before SCI, improved motor function by improving blood-spinal cord barrier integrity and NF-*κ*B decrement [344]. In a recent *in vivo* study on SCI by Subbarayan et al., treatment with human gingival-derived neural stem cells transplanted in the injectable caffeic acid bioconjugated hydrogel was associated with decreased IL-6 and TNF-*α* and increased IL-10; it was able to regenerate damaged spinal cord [327].

**Figure 3:**
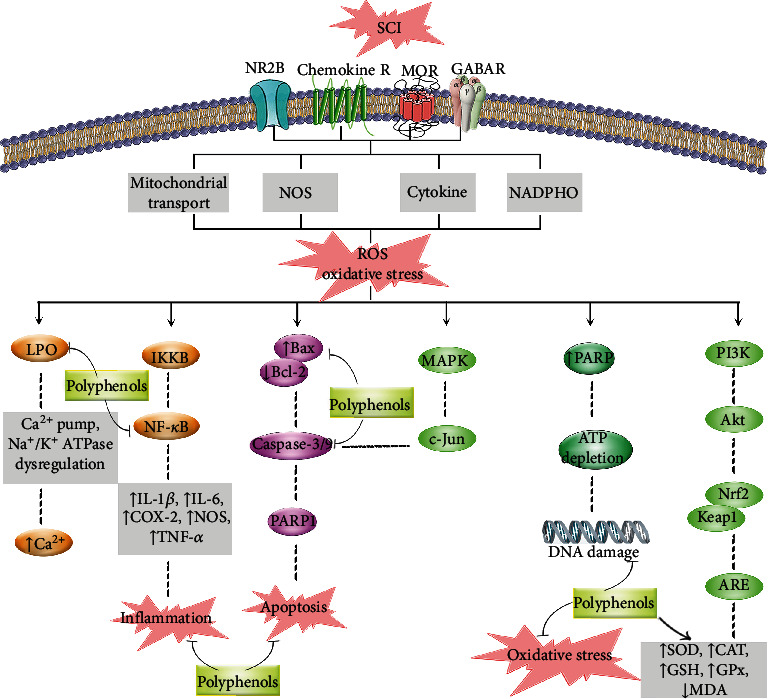
Oxidative stress and cross-talk signaling mediators post-SCI. The modulatory role of polyphenols. Akt: tyrosine kinase B; ARE: antioxidant response element; Ca^2+^: calcium; CAT: catalase; COX: cyclooxygenase; GABAR: gamma-aminobutyric acid receptor; GSH: glutathione; GPx: glutathione peroxidase; IKKB: IkB kinase; IL: interleukin; Keap1: Kelch-like ECH-associated protein 1; LPO: lipid peroxidation; MAPK: mitogen-activated protein kinase; MDA: malondialdehyde; MOR: morphine receptor; NADPHO: nicotinamide adenine dinucleotide phosphate oxidase; Na^+^/K^+^: sodium/potassium; NF-*κ*B: nuclear factor kappa-light-chain-enhancer of activated B; NOS: nitric oxide synthase; NR2B: N-methyl-D-aspartate receptor subtype 2B; Nrf2: nuclear factor erythroid 2-related factor 2; PARP: poly(ADP-ribose) polymerase; PI3K: phosphoinositide 3-kinase; SOD: superoxide dismutase; SCI: spinal cord injury; TNF-*α*: tumor necrosis factor-alpha.

Figure 3 demonstrates the major targets of polyphenols in combating oxidative stress, inflammation, and cross-linked pathways after SCI.

## 9. Clinical Potentials of Polyphenols against Oxidative/Inflammatory Disorders

Polyphenols are promising secondary metabolites with various health benefits. Curcuminoids have been approved by the US Food and Drug Administration (FDA) as generally safe and well-tolerated active metabolites in clinical trials, even in doses up to 12,000 mg/day [345].

Medicinal uses of curcumin have been reported with significant effects in protecting the liver and improving gastrointestinal function, respiration, cardiovascular, anti-inflammatory, anticancer, antidiabetic, antiallergic, neuroprotective, and chemoprotective [346] activities. A randomized, double-blind, placebo-controlled (RDBPC) study with 108 male adults aged between 31 and 59 years reported that curcumin in a dose of 1000 mg/day for 6 weeks (p.o.) decreased TNF-*α* and IL-1*β* levels and increased plasma BDNF [347].

In a controlled clinical trial study, the effects of curcumin on inflammatory and stress markers were evaluated in 100 patients of both sexes with osteoarthritis. Those studies showed that administration of curcumin in the dose of 200 mg/day for 8 months (p.o.) reduced inflammatory markers IL-1*β*, IL-6, and soluble ligand CD40 (sCD40L) [348]. In another study, prospective randomized open-end blinded evaluation (PROBE) on 80 patients with knee osteoarthritis, 30 mg/3 times in a day of curcumin (p.o.) for four weeks, reduced COX-2 [349]. Panahi et al. also reported that curcumin (1500 mg/day for six weeks) decreased levels of systemic inflammatory mediators, such as TNF-*α*, TGF-*β*, IL-6, substance P, and calcitonin gene peptide (CGRP) [350]. Another RDBPC study confirms the anti-inflammatory potential of oral curcumin (400 mg/3 times a day, p.o.) in patients with type 2 diabetes, along with a significant reduction in the levels of MDA, IL-6, and TNF-*α* [351].

In a controlled clinical trial study on 100 patients with SCI, curcumin (110 mg/day for six months) was notably associated with reducing osteoporosis progression and bone turnover markers after six months [352]. Results of a randomized, parallel-group, controlled, clinical trial on 20 subjects showed that administration of the InflanNox capsule (curcumin 1200 mg/day), with other anti-inflammatory and antioxidant effects, targeted inflammatory pathways, reduced IL-1*β*, and improved mood disorders in SCI patients [353]. Administration of nanocurcumin (80 mg/day) in 50 patients with multiple sclerosis was associated with a significant increase in the levels of TGF-*β* and IL-10 expression [354]. In another study, nanocurcumin was also employed in a randomized, double-blind, placebo-controlled clinical trial of 40 diabetic patients. In their study, nanocurcumin could suppress oxidative stress and free radicals as an antioxidant agent [355].

During a 52-week study, resveratrol in the dose of 500-1000 mg/day markedly modulated neuroinflammation, decreased MMP-9, and induced adaptive immunity [356]. Administration of 800 mg/day resveratrol for eight weeks showed an antioxidant effect in the serum of patients with type 2 diabetes. Expression of SOD, Nrf2, and plasma total antioxidant capacity was significantly increased [357]. Combined supplementation with resveratrol (80 mg/day) and EGCG (282 mg/day) for 12 weeks increased mitochondrial capacity and fat oxidation in obese humans [358]. As provided by Hendouei et al., resveratrol plus risperidone reduced irritability of patients with autism-associated oxidative stress in a RDBPC trial [359]. An open-label trial also confirmed the health benefits of high-dose resveratrol (1 g and 5 g/day) in 24 patients [360].

In an 8-month multicenter, RDBC trial, polyphenol supplementation (200 mL/day) potentially modulated plasma concentration of homocysteine in 48 patients with Alzheimer's disease [361]. In a multicenter, double-blind clinical study, 34 diabetic patients with neuropathy (21-72 years old) were subjected to receive a topical formulation containing quercetin to decrease oxidative stress [362]. Regarding evaluating the therapeutic effects of a herbal, polyphenol-rich extract in a randomized controlled trial, Verlaet et al. reported antioxidant potentials for attention-deficit hyperactivity disorder [363]. Besides, foods high in polyphenols showed the potential to speed up the cognitive reserve randomized factorial design with 180 participants with Alzheimer's disease [364]. Additionally, another polyphenol-riched extract showed potential antioxidative effects in healthy individuals and those subjected to neuronal-associated diseases [365–369].

We have also previously reported the clinical potential of the whole phytochemicals against the apoptotic/autophagy aspects of SCI and related neuronal dysfunctionalities [3].  Table 3 shows the clinical potentials of some polyphenols against oxidative/inflammatory-based disorders with the base of oxidative damage.

## 10. Conclusion

Oxidative stress seems to orchestrate the complex pathophysiological mechanisms in SCI regarding modulating other interconnected pathways, as neuroinflammation and neuroapoptosis. Consequently, there is a complicated cross-talk between oxidative stress and neuroinflammatory/neuroapoptotic pathways. In this line, Nrf2/Keap1/ARE, SOD, CAT, GSH, MDA, HO-1, and XO have dramatically attenuated the interconnected pathways/mediators, including Bax, Bcl-2, caspases, p53, and TNF-*α*, IL-1*β*, IL-6, and NF-*κ*B, toward neuroprotection in NDDs and SCI. The aforementioned signaling mediators are critically interconnected with extrinsic molecular mechanisms of regeneration post-SCI, including Nogo signaling pathways, glial scar formation, and chondroitin sulfate proteoglycan toward axonal activity. We also previously reported the complex dysregulated apoptotic and autophagic pathways following SCI (3). The complexity of those destructive signaling pathways of SCI and their interconnections urges the need to find multitarget agents with more tolerability and safety. In this regard, the plant kingdom has shown to be a promising source of neuroprotective agents. Growing studies are focusing on the potential of polyphenols in targeting oxidative stress and the cross-talk mediators (Figure 3). Our previous study also revealed the potential of polyphenols in targeting apoptosis and autophagy post-SCI (3). Additionally, it is worth noting that there are several common pathophysiological mechanisms behind the SCI and other NDDs. So, providing alternative therapeutic candidates for SCI could pave the way to treat other NDDs [372–374].

However, the polyphenol's pharmacokinetic limitations force the researchers to use novel drug delivery systems such as nanoformulations to delete such limitations in the clinical trial (3). So, the aforementioned limitation, along with the beneficial role of nanoparticles in improving the spinal cord drug delivery, proposes using nanoformulations of polyphenols to drawback such limitations. It will improve the beneficial effects of these compounds in SCI and other NDDs. Accordingly, metal nanoparticles (iron oxide, gold, silver, etc.), PLGA, polycaprolactone (PCL), poly-L-lactic acid (PLLA), liposomes, and inorganics are used toward the development of various nanoparticles in combating SCI complications [375]. Using novel drug delivery systems for phenolic compounds in SCI therapy could improve their bioavailability and facilitate the passages through the blood-spinal cord barrier and also modulate the water solubility and their long-lasting half-life following the spinal cord lesion [376].

Similar reviews will help the scientific community, neurosurgeons, neurooncologists, and patients to become aware of the complexity of dysregulated signaling pathways following SCI and the importance of finding novel multitarget alternative natural agents with more safety and efficacy. Further reports are needed to reveal the precise pathophysiological mechanisms and signaling pathways involved in NDDs, as well as the secondary phase of SCI. We propose targeting the upstream destructive mediators to prevent other interconnected pathways. The Nrf2, Keap1, and ARE seem to be hopeful candidates in the oxidative pathway to stop related pathogenicity. In this line, natural phytochemicals are proposed to be focused on as alternative treatments with lower side effects and higher efficacy. Among natural entities, polyphenols/phenolic compounds are considered secondary metabolites with extensive biological activities and health benefits, which are currently used in modern medicine toward designing and developing new therapeutic agents. Ongoing clinical trials are assessing the beneficial role of polyphenols on NDDs; however, there is a lack of clinical studies to evaluate the possible potential of polyphenols in combating post-SCI complications. So, well-controlled clinical trials will help reveal polyphenols' clinical potential in combating sensory-motor dysfunction following SCI and paving the road to overcome the possible limitations in their administration. In the present review, we highlighted the modulatory role of oxidative stress on the inflammatory and apoptotic cascades of NDDs, while tackling particular attention to SCI. We also focused our attention on the need to develop polyphenols/phenolic compounds as novel multitarget neuroprotective treatment through targeting the intrinsic oxidative mediators and inflammatory/apoptotic pathways, as well as extrinsic axonal associated pathways. The coadministration of polyphenols/phenolic compounds and stem cells could also pave the road in treating SCI complications. Such studies will provide novel therapeutic targets in the prevention, management, and treatment of NDDs and SCI and investigate polyphenols/phenolic compounds as promising phytochemicals. Future studies should focus on additional large-scale, well-controlled clinical trials on the administration of polyphenols/phenolic compounds alone or in combination with other phytochemicals, or stem cells/other drugs, concentrating on the intrinsic and extrinsic pathways of axonal regeneration.

## Figures and Tables

**Table 1:** Some preclinical studies on the effects of polyphenols in targeting oxidative stress and close interconnected mediators following SCI.

Phytochemicals	Dose	Pharmacological mechanisms	Animal models	References
Mangiferin	20, 40 mg/kg (i.p.); until 30 days post-SCI	↓ MDA; TNF-*α*; IL-1*β*; NF-*κ*B ↑ SOD; GPx; CAT	SD male rats; contusion-SCI; T10; 10 g × 12.5 mm	[19]
	10, 25, 50 mg/kg (i.p.); until 30 days post-SCI	↓ MDA; NF-*κ*B; TNF-*α*; IL-1*β*; IL-6; caspase-3; caspase-9 ↑ CAT; SOD; GSH	SD rats; contusion-SCI; T8-T10; 6 g × 100 mm	[256]
Quercetin	0.2 mg/kg (i.p.); 1 h post-SCI	↓ iNOS; p38MAPK; MDA; SOD	SD male rats; contusion-SCI; T10; 5 g × 80 mm	[61]
	0.25 *μ*mol/kg (i.p.); 1 h post-SCI	↓ MPO	Wistar male rats; compression-SCI; T6-T7; 70 g force; 5 s	[72]
	10, 100 mg/kg (i.p.); first 72 h post-SCI	↓ MDA; NO	Wistar male rats; compression-SCI; T7-T9; 70 g force 1 min	[73]
	20 mg/kg (i.p.); twice daily until 7 days post-SCI	↓ MDA; MPO; NO; IL-1*β*; IL-6; TNF-*α*; caspase-3 ↑ SOD; GSH	Wistar albino rats; contusion-SCI; T7-T10; 10 g × 100 mm	[75]
	7.5 mg/kg (i.p.); twice daily until 10 days post-SCI	↓ TNF-*α*; IL-1*β*; p-STAT1; iNOS; NF-*κ*B; IL-12 ↑ IL-4; IL-10; TGF-*β*	SD male rats; compression-SCI; T8	[77]
	100 mg/kg (i.p.); until 3 days post-SCI	↓ ROS; IL-1*β*; IL-18; TNF-*α*	SD female rats; compression-SCI; T10	[78]
EGCG	50 mg/kg (i.p.); immediately and 1 h post-SCI	↓ iNOS; MPO; COX-2; TNF-*α*; IL-1*β*; PARP	SD male rats	[89]
	30 mg/kg (i.p.); until 7 days post-SCI	↓ TNF-*α*; RhoA	Female BALB/c mice; contusion-SCI; T8-T9; 2 g × 25 mm	[93]
Protocatechuic acid	50 mg/kg (i.p.)	↓ TNF-*α*; IL-1*β*; COX-2; iNOS; MMP-9	SD male rats; contusion-SCI; T9-T10; 10 g × 25 mm	[94]
Caffeic acid phenethyl ester	10 *μ*L; 1 *μ*g/kg (i.t.); 1 h post-SCI	↓ MDA; SOD; TOA ↑ TAC	Wistar female mice; compression-SCI; T5-T12; 63 g force; 1 min	[109]
	10 *μ*g/kg (i.p.); 30 min post-SCI	↓ IL-1*β*; TNF-*α*	Wistar albino male rats; compression-SCI; T8-T12; 1.37-1.72 N force; 1 min	[111]
	10 *μ*mol/kg (i.p.); until 4 weeks post-SCI	↓ COX-2; NOS; IL-1*β*	Wistar female rat; hemitransection-SCI; T10	[112]
Honokiol	20 mg/kg (i.p.)	↓ MPO; iNOS; COX-2; IL-1*β*; IL-6; TNF-*α*	SD female rats; contusion-SCI; T10; 25 g/cm	[131]
Rutin	50, 100 mg/kg (i.p.); until 3 days post-SCI	↓ MDA; ROS; IL-1*β*; IL-18; TNF-*α*	SD female mice; compression-SCI; T9-T10	[161]
	30 mg/kg (i.p.); until 3 days post-SCI	↓ MDA; p38MAPK; IL-1*β*; IL-6; TNF-*α*; NF-*κ*B; ↑ SOD; GSH; CAT	SD male rats; contusion-SCI; T8-T9; 10 g × 50 mm	[171]
	30 mg/kg (i.p.); until 3 days	↓ TNF-*α*; MDA; ROS; TGF-*β*1; Smad2 ↑ MPO	Rats; contusion-SCI T10-T11; 12 g × 7 mm	[174]
Hesperidin	100 mg/kg; 7 days pre-SCI until 7 days post-SCI	↓ IL-1*β*; TNF-*α*; NF-*κ*B; PARP; ↑ SOD; CAT; Nrf2; HO-1; p-p38	SD female rats; compression-SCI; T9-T10; 20 g force; 1 min	[186]
Oxyresveratrol	10, 20 mg/kg (i.p.); until 4 weeks post-SCI	↓ MDA; IL-1*β*; IL-6; TNF-*α*; NF-*κ*B; COX-2; iNOS; ↑ Nrf2; SOD; GPx; GSH	SD female rats; contusion-SCI; T10; 10 g × 25 mm	[206]
Polydatin	20, 40 mg/kg (i.p.); 30 min post-SCI	↓ MDA; NO; iNOS; IL-1*β*; IL-6; TNF-*α*; NF-*κ*B; ↑ SOD; Nrf2; HO-1	SD male rats; contusion-SCI; T8; 10 g × 50 mm	[208, 209]
Resveratrol	100 mg/kg (i.p.); immediately post-SCI	↓ MDA; NO; XO; ↑ GSH	Wistar male rats; contusion-SCI; T7-T10; 5 g × 100 mm	[201]
	100 mg/kg (i.p.); until 2 weeks post-SCI	↓ Caspase-3; ↑ LC3; Beclin1; PI3K	SD rats; contusion-SCI; T9-T10	[211]
	10 mg/kg (i.p.)	↓ MDA; p38MAPK; ↑ SOD; CAT; GSH	Wistar male rats; Spinal cord ischemia-reperfusion injury	[214]
	1 and 10 mg/kg; 30 min pre-SCI	↓ NO; MDA	Rabbits, spinal cord ischemia-reperfusion injury	[215]
	400 mg/kg (p.o.); until 10 days post-SCI	↓ MDA; IL-6 ↑ TCA	SD male rats; transection-SCI T10-T12	[216]
	50, 100 mg/kg (i.p.); immediately post-SCI	↓ MDA; ↑ Na^+^, K^+^-ATPase activities	SD male and female rats; contusion-SCI; T8; 10 g × 25 mm	[217]
	200 mg/kg (i.p.); until 3 days post-SCI	↓ MDA; MPO; IL-1*β*; IL-10; TNF-*α*; ↑ SOD	SD rats; contusion-SCI; T10; 10 g × 25 mm	[285]
Curcumin	50, 100, 200 mg/kg (i.v.); until 7 days post-SCI	↓ p38MAPK; NF-*κ*B	Female mice; contusion-SCI; T9-T10; 3 g × 30 mm	[229]
	100 mg/kg (i.p.)	↓ MDA; NO; TBARS	Long Evans female rats; contusion-SCI; 10 g × 25 mm	[231]
	200 mg/kg (i.p.); immediately post-SCI	↑ SOD; GPx; CAT	Wistar male rats; compression-SCI; T7; 70 g force; 1 min	[232]
	300 mg/kg (i.p.)	↓ MDA	Wistar albino female rats; contusion-SCI; T7-T9; 5 g × 100 mm	[233]
	200 mg/kg (i.p.); until 8 weeks post-SCI	↓ MDA; IL-6; IL-8; TNF-*α*; p-ERK; p-p38; p-JNK; p-STAT; GFAP; ↑ SOD	SD female rats; compression-SCI; T9-T10; 30 g force; 2 min	[234]
	30 mg/kg (i.p.); until 7 days post-SCI	↓ NMDA; iNOS	SD rats; ischemic SCI	[235]
	50 mg/kg (i.p.); until 7 days post-SCI	↓ IL-1*β*; NO; NF-*κ*B; STAT3	BALB/c female mice; compression-SCI; T8-T9; 30 g force, 1 min	[236]
	40 mg/kg (i.p.); 30 min post-SCI	↓ MDA; iNOS	SD male rats; contusion-SCI; T9-T10; 10 g × 25 mm	[237]
	300, 100, 30 mg/kg (i.p.); until 7 days post-SCI	↓ TNF-*α*; IL-1*β*; NF-*κ*B; TGF-*β*1; TGF-*β*2	SD male rats; compression-SCI; T8-T10; 50 g force; 1 min	[238]
	40 mg/kg (i.p.); 30 min post-SCI	↓ iNOS; ↑ Bcl-2, CISD2	C57BL/6JNarl mice; hemisection and contusion-SCI; T9-T10; 10 g × 25 mm	[239]
	100 mg/kg (i.p.); until 7 days post-SCI	↓ SOX9; NF-*κ*B; GFAP; CD45; CD11b	SD female rats; compression-SCI; T8-T10; 50 g force; 1 min	[240]
	60, 6 mg/kg (i.p.); until 4 weeks post-SCI	↓ IL-4; IL-1*β*; IL-2; IL-6; IL-12; TNF-*α*; MIP-1*α*; NF-*κ*B; GFAP	Wistar male rats; balloon compression-SCI; T8-T10	[241]
	40 mg/kg (i.p.); 30 min post-SCI	↓ GFAP; ↑ p-JAK2; p-STAT3	SD male rats; contusion-SCI; T8	[242]
	75, 150, 300 mg/kg (i.p.); 20 min and until 3 days post-SCI	↓ TNF-*α*; NF-*κ*B	SD male rats; compression-SCI; T8-T10	[243]
	100 mg/kg (i.p.); 15 min post-SCI	↓ TNF-*α*; IL-1*β*; NF-*κ*B; TLR4	SD male rats; compression-SCI T8-T9; 30 g force	[244]
	200 mg/kg (p.o.)	↓ MDA; ↑ SOD	Wistar albino male rats; contusion-SCI T7-T10; 5 g × 100 mm	[286]
	200 mg/kg (i.p.); until 7 days post-SCI	↓ MDA; ↑ SOD	SD male rats; contusion-SCI T8	[287]
	60 mg/kg (i.p.); 30 min and weekly for 3 weeks post-SCI	↓ Caspase-3; IL-6; IL-1*β*; TNF-*α*; p-mTOR; p-Akt; Iba1; GFAP ↑ LC3	SD male rats; contusion-SCI; T9-T11; 10 g × 20 mm	[288]
	200 mg/kg (i.p.); until 8 weeks post-SCI	↓ Caspase-3; Bax; GFAP; ↑ Bcl-2	SD male rats; contusion-SCI; T9-T10; 10 g × 12.5 mm	[289]
Apocynin	50 mg/kg (i.p.); twice daily until 3 days post-SCI	↓ MPO; MDA; TNF-*α*; IL-1*β*; IL-6; ↑ GSH; SOD	SD male rats; compression-SCI; T6-T7; 24 g force; 1 min	[268]
Baicalin	50, 100 mg/kg (p.o.)	↓ MDA; IL-1*β*; IL-6; TNF-*α*; NF-*κ*B; ↑ SOD; GPx; CAT	SD male mice; compression-SCI; T7; 23.8 g force; 2 min	[270]
	10, 30, 100 mg/kg (i.p.); immediately and 24 h post-SCI	↓ MDA; NF-*κ*B; TNF-*α*; Bax; ↑ GSH; Bcl-2	SD male rats; contusion-SCI; T12; 50 g	[271]
Gastrodin	100, 200 mg/kg (i.p.); until 5 days post-SCI	↓ TBARS ↑ SOD; GSH; Nrf2; GCLc; GCLm	SD male rats; contusion-SCI; T10; 8 g × 30 mm	[272]
Mulberrin	15, 30 mg/kg (p.o.); until 4weeks post-SCI	↓ TBARS; ROS; IL-1*β*; IL-6; TNF-*α*; NF-*κ*B; PARP; ↑ SOD; GSH; Nrf2; HO-1	SD male rats; compression-SCI; T10; 30 g force; 1 min	[273]
Salvianolic acid A	2.5, 5, 10 mg/kg (i.p.); until 7 days post-SCI	↑ Nrf2; HO-1	SD male rats; compression-SCI; T12; 50 g force; 5 min	[274]
Salvianolic acid B	1, 10, 50 mg/kg (i.p.) until 3 days post-SCI	↓ TNF-*α*; NF-*κ*B; ↑ HO-1	SD male rats; compression-SCI; T12; 50 g force; 5 min	[275]
Gallic acid	50 mg/kg (i.p.); immediately, 6 h and 12 h, and then one day until 7 days post-SCI	↓ MMP-9; TNF-*α*; IL-1*β*; IL-6; COX-2; iNOS	SD rats; contusion-SCI; T9-T10; 10 g × 25 mm	[276]
	10 mg/kg (i.p.); until 10 days post-SCI	↓ COX-2; NF-*κ*B	Wistar male rats; contusion-SCI; T9; 15 g × 25 mm	[277]
Kaempferol		↓ IL-18; IL-1*β*; ROS; p-p38MAPK; p-JNK; NF-*κ*B	SD rat; hemicontusion-SCI	[278]
Luteolin and palmitoylethanolamide	1 mg/kg (i.p.); 1 and 6 hours post-SCI 27 and 2.7 *μ*M	↓ nNOS; iNOS; COX-2	CD1 mice; compression-SCI; T5-T8; 24 g force; *in vitro*: spinal cord slices	[279]
Naringenin	50, 100 mg/kg (p.o.); 3 days pre-SCI until 7 days post-SCI	↓ TNF-*α*; IL8; IL-1*β*; IL-6	SD male rats; compression-SCI T9-T11	[280]
Oleuropein	20 mg/kg (i.p.); immediately and 1 h post-SCI	↓ MDA; Bax; ↑ Bcl-2; GSH	SD female rats; contusion-SCI; T9; 10 g × 25 mm	[281]
	250 *μ*L at the dose of 20/40/100 *μ*g/kg (i.p.); 1 h and 6 h post-SCI	↓ MPO; IL-1*β*; TNF-*α*; NF-*κ*B; LPO; TBARS; ↑ GDNF	CD1 male mice; compression-SCI; T5-T8; 24 g; 1 min	[282]
Pycnogenol	100 mg/kg (i.p.); 15 min post-SCI	↓ Bax; caspase-3; MDA; ↑ Bcl-2; SOD; MMP	Wistar male rats; contusion-SCI; T10; 10 g × 50 mm	[283]
Silymarin	Injected 1–2 mm caudal and rostral to the epicenter; 5 min post-SCI	↓ ROS; NF-*κ*B; COX-2; iNOS; IL-1*β*	SD rats; contusion-SCI; T9-T10; 10 g × 50 mm	[284]
Carnosol	5 mg/kg (i.p.); 1 h post-SCI	↓ COX-2; NF-*κ*B; IL-1*β*; IL-6; TNF-*α*; ↑ TAC; GSH; GPx; GST; SOD; CAT; p-Akt; Nrf2	SD female rats; contusion-SCI; T7-T10; 10 g × 100 mm	[290]
Tetrahydrocurcumin	80 mg/kg (i.p.); until 2 weeks post-SCI	↓ MDA; TNF-*α*; IL-6; IL-1*β*; NF-*κ*B; ↑ SOD; GSH; GPx; p-Akt; FoxO-4	SD male rats; compression-SCI; T8; 30 g force; 2 min	[291]

Akt: tyrosine kinase B; Bax: Bcl-2-associated X protein; Bcl: B cell lymphoma; caspases: cysteine-dependent aspartate-directed proteases; CAT: catalase; COX: cyclooxygenase; eNOS: endothelial nitric oxide synthase; ERK: extracellular signal-regulated kinases; FoxO-4: forkhead box O4; GCLc: catalytic subunit of glutamate-cysteine ligase; GCLm: modifying subunit of glutamate-cysteine ligase; GFAP: glial fibrillary acidic protein; GSH: glutathione; GST: glutathione S-transferase; GPx: glutathione peroxidase; HO-1: heme oxygenase-1; Iba1: ionized calcium-binding adaptor molecule 1; IL: interleukin; iNOS: inducible nitric oxide synthase; i.p.: intraperitoneal; i.t.: intrathecal; i.v.: intravenous; JAKs: Janus kinases; JNK: c-Jun N-terminal kinase; LC3: light chain 3; LPO: lipid peroxidation; MAPK: mitogen-activated protein kinase; MDA: malondialdehyde; MIP-1*α*: macrophage inflammatory protein 1-alpha; MPO: myeloperoxidase; mTOR: mammalian target of rapamycin; NADPH oxidase: nicotinamide adenine dinucleotide phosphate; NF-*κ*B: nuclear factor kappa B; NO: nitric oxide; Nrf2: nuclear factor erythroid 2-related factor 2; PARP: poly(ADP-ribose) polymerase; p.o.: oral administration; ROS: reactive oxygen species; SCI: spinal cord injury; SD: Sprague Dawley; SOD: superoxide dismutase; SOX9: SRY-box transcription factor 9; STATs: signal transducer and activator of transcription proteins; T: thoracic; TAC: total antioxidant capacity; TBARS: thiobarbituric acid reactive substance; TLR4: transmembrane lipopolysaccharide receptor; TNF-*α*: tumor necrosis factor-alpha; XO: xanthine oxidase.

**Table 2:** Targeting extrinsic regenerative/degenerative mechanisms by polyphenols post-SCI.

Phytochemicals	Dose	Pharmacological effects	Animal models	References
Quercetin	20 mg/kg (i.p.); until 7 days post-SCI	↓ Cavity formation; glial scar (GFAP); ↑ axonal regeneration (5-HT; NF200); locomotor recovery	SD male rats; contusion-SCI T8; 50 g × cm	[71]
	7.5 mg/kg (i.p.); twice daily until 10 days post-SCI	↑ Locomotor recovery; preventing necroptosis; preservation of myelin and axonal	SD male rats; compression-SCI T8	[77]
EGCG	50 mg/kg (directly to the spinal cord surface) until 4 weeks post-SCI	↓ Glial scar (GFAP); ↑ growth factors (FGF2; VEGF); axonal sprouting (GAP43); locomotor recovery; preservation of white and grey matter; modulation macrophage markers (M1 and M2)	Wistar rats; balloon compression-SCI T8	[82]
Curcumin and EGCG	60 mg/kg 17 mg/kg (i.p.); until 4 weeks post-SCI	↓ Glial scar (GFAP); ↑ axonal sprouting (GAP43; Olig2); locomotor recovery; tissue regeneration; neuroprotective effects	Wistar male rats; balloon compression-SCI T10	[92]
EGCG	30 mg/kg (i.p.); until 7 days post-SCI	↓ TNF-*α*; RhoA; glial scar (GFAP) ↑ Sensory recovery	Female BALB/c mice; contusion-SCI T8-T9; 2 g × 25 mm	[93]
Curcumin	60, 6 mg/kg (i.p.); until 4 weeks post-SCI	↓ Glial scar (GFAP); ↑ axonal sprouting (GAP43; Olig2); locomotor recovery Anti-inflammatory effects	Wistar male rats; balloon compression-SCI T8-T10	[241]
Polymer-curcumin and ependymal progenitor/stem cell	405 mg; 1.1 mmol	↓ ROCK1; RhoA; GAP43; p-Limk1; glial scar (GFAP); microglial activation (Iba1); macrophage infiltration (ED1); ↑ locomotor recovery	SD rats; Horizon Impactor-SCI T8; 250 kdyn	[309]
Nanoformulated curcumin	0.01 mL	↓ GAP43; IL-1*β*; TNF-*α*; IFN-*γ*; IL-6; glial scar; ↑ functional recovery; preservation of white matter	Wistar male rats; balloon compression-SCI T10	[322]
Curcumin and mesenchymal stem cells	60, 6 mg/kg (i.p.); until 4 weeks post-SCI	↓ Glial scar (GFAP); ↑ axonal sprouting (GAP43); locomotor and sensory recovery; preservation of white and grey matter	Wistar rats; balloon compression-SCI T8	[323]
Naringin	20, 40 mg/kg (p.o.); until 4 weeks post-SCI	↑ Remyelination (GSK-3*β*; *β*-catenin); axonal sprouting (NG2; Olig2); locomotor recovery	SD female rats; contusion-SCI T10	[326]
Caffeic acid hydrogel and human gingival-derived neural stem cell		↓ Glial scar (GFAP); ↑ axonal growth; recovery of damaged spinal tissue; locomotor recovery Maturation of neuronal cells	Wistar female rats; hemitransection-SCI T9-T11	[327]

EGCG: epigallocatechin gallate; GAP43: Growth Associated Protein 43; GFAP: glial fibrillary acidic protein; GNSCs: human gingival-derived neural stem cell; GSK3B: glycogen synthase kinase-3 beta; Iba1: ionized calcium-binding adaptor molecule 1; i.p.: intraperitoneal; Olig2: oligodendrocytes; p.o.: oral administration; ROCK1: Rho-associated coiled-coil containing protein kinase 1; SCI: spinal cord injury; SD: Sprague Dawley; T: thoracic; TGF-*β*: transforming growth factor-beta; TNF-*α*: tumor necrosis factor-alpha; VEGF: vascular endothelial growth factor.

**Table 3:** Clinical potentials of polyphenols against oxidative/inflammatory disorders.

Polyphenols	Clinical study	Population	Protocol	Therapeutic findings	References
Curcumin	Major depressive disorder DBRPC	*N* = 108; *M*; age = 31-59	1000 mg/day of curcumin/soybean powder; 6 weeks	↓ TNF-*α* and IL-1*β*; ↑ BDNF	[347]
	Osteoarthritis Controlled clinical trial	*N* = 100 Treatment (50): *M* = 23 *W* = 27 Age = 43.6 Control (50): *M* = 28 *W* = 22 Age = 44.2	1000 mg/day of Meriva (containing 200 mg curcumin); 32 weeks	↓ IL-1*β* and IL-6	[348]
	Osteoarthritis Randomized controlled trial	*N* = 80 Curcuminoid (39): *M* = 15 *W* = 24 Diclofenac (41): *M* = 12 *W* = 29	30 mg of curcuminoid; 25 mg of diclofenac sodium; 3 times daily, 4 weeks	↓ COX-2	[349]
	Osteoarthritis RBDCT	*N* = 53 Curcuminoid (19): *M* = 5 *W* = 14 Age = 57.32 Placebo (21): *M* = 4 *W* = 17 Age = 57.57	1500 mg/day of curcuminoid/placebo; 6 weeks	↓ TNF-*α*, TGF-*β*, IL-6, substance P, and CGRP	[350]
	Type 2 diabetes Clinical trial		400 mg/3 times a day	↓ MDA, IL-6, and TNF-*α*	[351]
	SCI Randomized clinical trial	*N* = 20; age = 30-67 Treatment (12): *M* = 6 *W* = 6 Control (8): *M* = 4 *W* = 4	Omega-3 250-500 mg/3 times a day; Chlorella 1000 mg/6 times a day; antioxidants (100 mg coenzyme Q10, 200 mg N-acetylcysteine, 150 mg mixed tocopherols, 100 mg DL-alpha-lipoic acid, 60 mg green tea extract, 5.5 mg zinc, and 100 *μ*g selenium, 2 times a day); curcumin 400 mg/3 times a day; 12 weeks	↓ IL-1*β*	[353]
	Multiple sclerosis Randomized controlled trial	*N* = 50 Curcuminoid (25): *M* = 9 *W* = 16 Age = 35.2 Placebo (25): *M* = 10 *W* = 15 Age = 34.6	80 mg/day of nanocurcumin; 24 weeks	↓ TGF-*β* and IL-10	[354]
	Metabolic syndrome Randomized controlled trial	*N* = 117 Curcuminoid (58): *M* = 35 *W* = 23 Age = 44.80 Placebo (59): *M* = 32 *W* = 27 Age = 43.46	1000 mg/day of curcuminoid/placebo; 8 weeks	↓ MDA, TNF-*α*, TGF-*β*, and IL-6; ↑ SOD	[370, 371]
Resveratrol	Alzheimer's disease RBDCT	*N* = 119 Resveratrol (56) Placebo (48)	500 mg/day of placebo/resveratrol (with a dose escalation by 500 mg increments every 13 weeks, ending with 1000 mg twice daily) 52 weeks	↓ MMP-9; ↑ MMP-10; FGF-2 and IL-4	[356]
	Type 2 diabetes RBDCT	*N* = 41 Resveratrol (23): *M* = 11 *W* = 12 Age = 54.96 Placebo (18): *M* = 8 *W* = 10 Age = 58.72	800 mg/day of resveratrol/placebo; 8 weeks	↑ TAC; Nrf2 and SOD	[357]
	Obesity RBDCT	*N* = 42; *M* = 21; *W* = 21 Treatment: Age = 36.1 Placebo: Age = 38.7	282 mg/day of EGCG and 80 mg/day of resveratrol/placebo; 12 weeks	↑ Mitochondrial capacity and fat oxidation	[358]

BDNF: brain-derived neurotrophic factor; CGRP: calcitonin gene peptide; COX: cyclooxygenase; DBRPC: double-blind, randomized, placebo-controlled; IL: interleukin; *M*: male; MDA: malondialdehyde; MMP: matrix metallopeptidase; *N*: number; Nrf2: nuclear factor erythroid 2-related factor 2; PROBE: prospective randomized open-end blinded evaluation, RBDCT: randomized double-blinded controlled trial; SCI: spinal cord injury; SOD: superoxide dismutase; TAC: total antioxidant capacity; TGF-*β*: transforming growth factor-beta; TNF-*α*: tumor necrosis factor-*α*; *W*: woman.
